# Quantitative RNA pseudouridine maps reveal multilayered translation control through plant rRNA, tRNA and mRNA pseudouridylation

**DOI:** 10.1038/s41477-024-01894-7

**Published:** 2025-01-09

**Authors:** Haoxuan Li, Guanqun Wang, Chang Ye, Zhongyu Zou, Bochen Jiang, Fan Yang, Kayla He, Chengwei Ju, Lisheng Zhang, Boyang Gao, Shun Liu, Yanming Chen, Jianhua Zhang, Chuan He

**Affiliations:** 1 Department of Chemistry, Department of Biochemistry and Molecular Biology, and Institute for Biophysical Dynamics, The University of Chicago, Chicago, IL, USA.; 2 Howard Hughes Medical Institute, Chicago, IL, USA.; 3 Department of Biology, Hong Kong Baptist University and School of Life Sciences and State Key Laboratory of Agrobiotechnology, The Chinese University of Hong Kong, Hong Kong, China.; 4 These authors contributed equally: Haoxuan Li, Guanqun Wang, Chang Ye.

## Abstract

Pseudouridine (Ψ) is the most abundant RNA modification, yet studies of Ψ have been hindered by a lack of robust methods to profile comprehensive Ψ maps. Here we utilize bisulfite-induced deletion sequencing to generate transcriptome-wide Ψ maps at single-base resolution across various plant species. Integrating ribosomal RNA, transfer RNA and messenger RNA Ψ stoichiometry with mRNA abundance and polysome profiling data, we uncover a multilayered regulation of translation efficiency through Ψ modifications. rRNA pseudouridylation could globally control translation, although the effects vary at different rRNA Ψ sites. Ψ in the tRNA T-arm loop shows strong positive correlations between Ψ stoichiometry and the translation efficiency of their respective codons. We observed a general inverse correlation between Ψ level and mRNA stability, but a positive correlation with translation efficiency in *Arabidopsis* seedlings. In conclusion, our study provides critical resources for Ψ research in plants and proposes prevalent translation regulation through rRNA, tRNA and mRNA pseudouridylation.

Pseudouridine (Ψ) is an isomerization of uridine (U)-to-pseudouridine in living organisms^1–3^. Ψ is abundant in various ribosomal RNAs (25S, 18S, 5.8S and 5S rRNAs), transfer RNA and small nucleolar RNA, with high levels ofconservation in yeast^4–7^ and mammals^8–11^. For example, Ψ located at position 55 in tRNA is an almost universally conserved RNA modification found in all three domains of life^12^. Relatively abundant Ψ in messenger RNA in mammals^10,11,13,14^ and *Arabidopsis*^15^ was recently revealed. Translation, one of the most important biological transformations, is not merely a simple flow of information from the transcriptome to the proteome mediated by ribosomes, involving many factors and playing important roles. For instance, tRNA can be a key regulator that guides translation rate and efficiency through codon usage and tRNA modifications. Pseudouridylation being the most abundant type of modification in tRNA ensures proper translation^16,17^. rRNA Ψ modification affects ribosome activity and modulates affinities for tRNA binding, leading to altered translational fidelity and translational initiation in yeast and human cells^18–22^. Ψ insertion into mRNA codons could perturb ribosome function and promote peptide synthesis, suggesting translation alternation by mRNA pseudouridylation in mammal cells^23^. Therefore, the effects of Ψ on translation are complex and involve multiple RNA species.

RNA pseudouridylation in plants and its effects on translation have yet to be studied adequately. In *Arabidopsis*, a pseudouridine synthase gene, *leaf curly and small 1* (*FCS1*), has been shown to affect fertility and development^24^, whereas the *SUPPRESSION OF VARIAGATION 1 (SVR1)* gene encodes a chloroplast-localized pseudouridine synthase that regulates chloroplast rRNA biogenesis^25^. Dysfunction of *OsPUS1*, the *SVR1* orthologue gene in rice, leads to aberrant chloroplast ribosome biogenesis and defective chloroplast development at low temperatures^26^. However, the functions of plant RNA Ψ have yet to be systematically explored, mostly because of a lack of effective methods to quantitatively map Ψ and of high-resolution RNA Ψ maps in different plant species.

Previous approaches to detect Ψ mostly used *N*-cyclohexyl-*N*′-(2-morpholinoethyl)carbodiimide methyl-*p*-toluenesulfonate (CMC) that modifies Ψ and blocks reverse transcription^8,10^. An azide-modified CMC has also been used to enrich Ψ-containing RNA fragments for transcriptome-wide Ψ mapping^11^. More recently, we and others have developed a bisulfite-induced deletion sequencing (BID-seq) method, based on a previous observation^27,28^, to uncover Ψ sites in mammal mRNA^13,29^. BID-seq^28^ and the related method of PRAISE^29^ could induce high deletion ratios at Ψ modification sites and have close to zero background deletions at unmodified uridines, thereby generating reproducible results with modification stoichiometry information. We therefore used this high-resolution sequencing method to establish comprehensive maps of non-coding RNA and mRNA Ψ sites at single-nucleotide resolution across various tissue types in four different plant species: *Arabidopsis*, rice, maize and soybean.

Our systematic investigation into Ψ modifications revealed previously unknown translation control pathways in plants. We found that although rRNA Ψ sites are conserved in different plants, the modification stoichiometry may globally affect translation depending on the locations of rRNA Ψ sites. Specifically, we observed that mRNA transcripts with longer 5′-untranslated regions (UTRs) showed more positive correlations between Ψ level and translation efficiency, particularly for the Ψ site of Nu-18S:913 in the ribosomal helix 22 initiation region. By contrast, mRNA transcripts with greater coding sequence (CDS) length showed more positive correlation between Ψ level and translation efficiency for the Ψ site of Nu-18S:1,195 in the ribosomal helix 31 decoding region. These observations suggest that translation of mRNAs possessing longer 5′-UTRs might be more strongly subjected to regulation through pseudouridylation at the initiation regions of rRNA, whereas mRNAs with longer CDS could be more strongly subjected to regulation through pseudouridylation at the decoding regions of rRNA. We also noticed a positive correlation between tRNA Ψ modification stoichiometry and translation efficiency of their respective codons in the tRNA loop region, but not the stem region, in *Arabidopsis*.

Our comparative analysis revealed that non-coding RNA Ψ sites and their modification fractions, but not mRNA Ψ sites, show high conservation between plant species. For instance, conserved rRNA regions tend to have higher densities of Ψ sites, with variable regions containing far fewer modification sites. This might suggest a mechanism that rRNA sequence conservation allows for region-specific Ψ modification. We further provide mRNA Ψ site maps of both rice and *Arabidopsis* in different tissues spanning the entire plant life cycle. We found that a large proportion of mRNA Ψ sites are tissue-specific both in *Arabidopsis* and rice, suggesting roles for these Ψ sites in the regulation of tissue differentiation and development in plants. Using base-resolution data, we noticed a generally negative correlation between Ψ level and the stability of the modified mRNA, and a positive correlation between Ψ level and its mRNA translation efficiency in *Arabidopsis* seedlings. Therefore, our quantitative mapping of Ψ stoichiometry on rRNA, tRNA and mRNA reveals a multilayered regulation of translation through pseudouridylation of these RNA species, with rRNA possessing conserved Ψ sites to affect more global translation, but also more strongly affect genes with longer 5′-UTR or CDS. The tRNA Ψ sites in loop regions, but not stem regions, might positively affect translation efficiency, and mRNA Ψ sites exert mRNA-specific translation regulation dependent on the location and stoichiometry of the modified site.

## Results

### BID-seq reveals conserved pseudouridylation in rRNA

**Extended Data Fig. 1 |:**
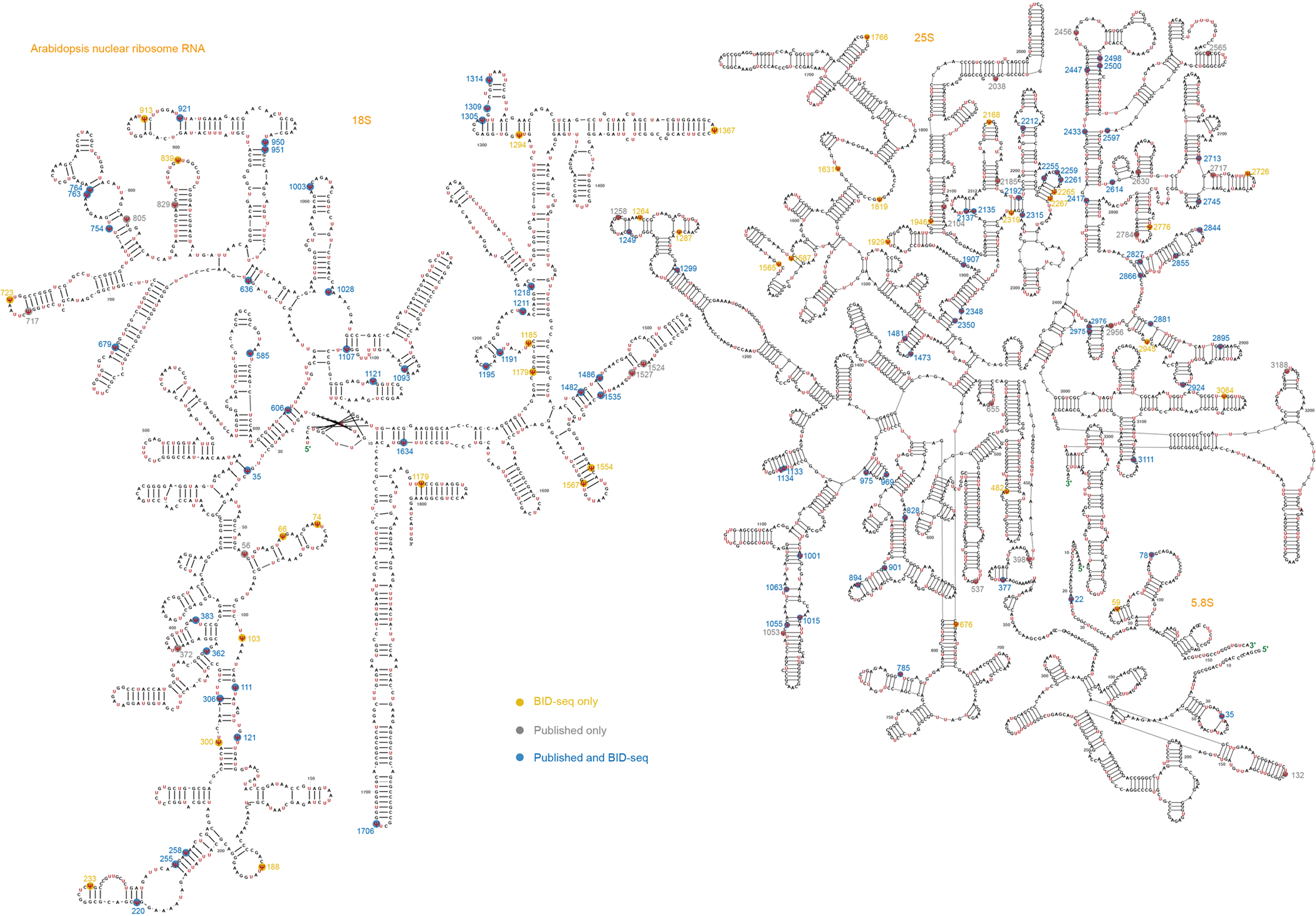
The rRNA Ψ sites in the rRNAs of nuclear 18S, nuclear 5.8S and nuclear 25S identified in *Arabidopsis* using BID-seq. The Ψ sites were accurately marked and compared with previously published sites. Yellow color marks the Ψ sites identified by BID-seq only; Grey color marks the Ψ sites only previously published, but not identified in this study; Blue color marks the Ψ sites that not only identified in this study but reported previously.

**Extended Data Fig. 2 |:**
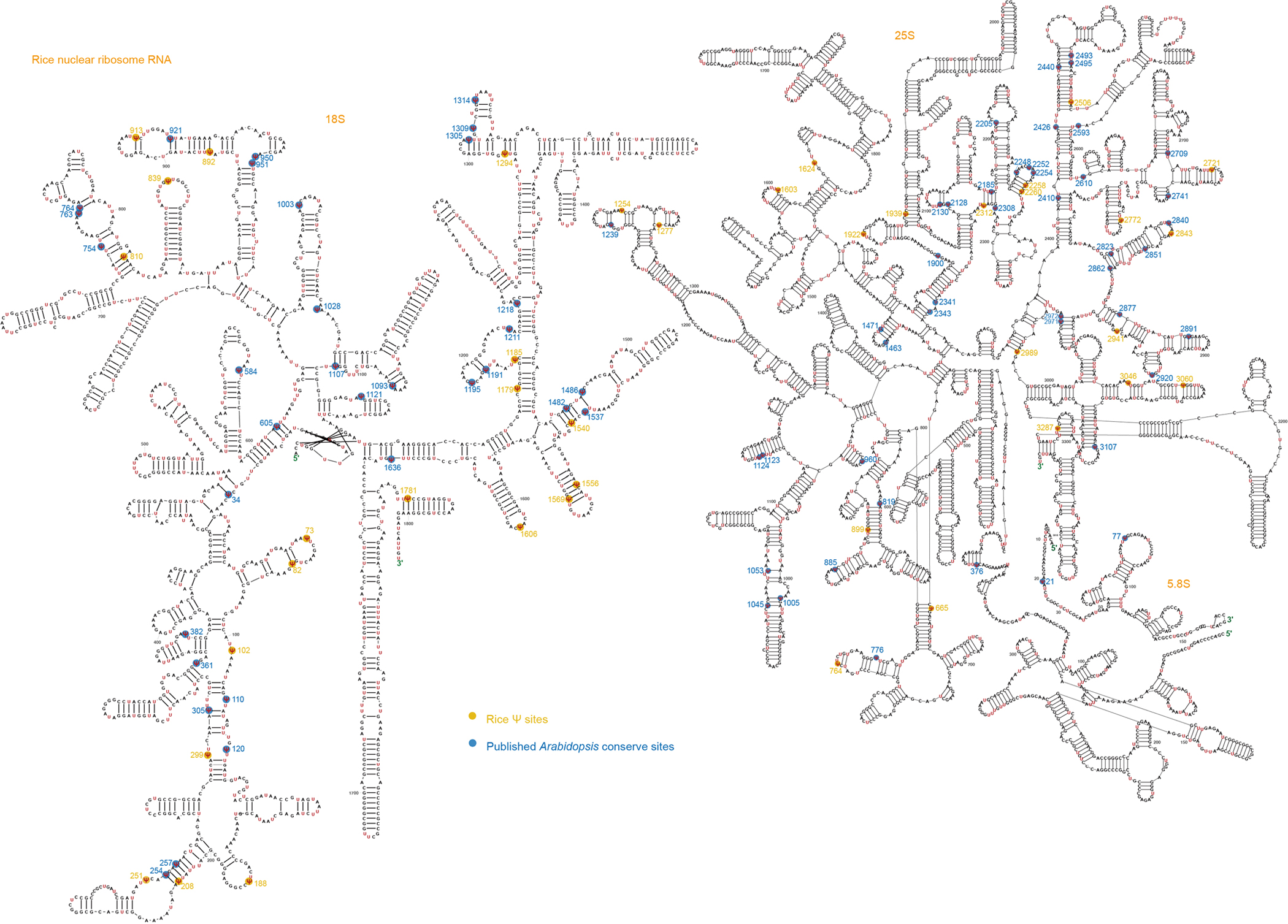
The conserved Ψ sites in the rRNAs of nuclear 18S, nuclear 5.8S and nuclear 25S in rice and *Arabidopsis.* The Ψ sites were accurately marked and compared with previously published sites. Yellow color marks the Ψ sites only in rice. Blue color marks the Ψ sites conserved in both rice and *Arabidopsis*.

**Extended Data Fig. 3 |:**
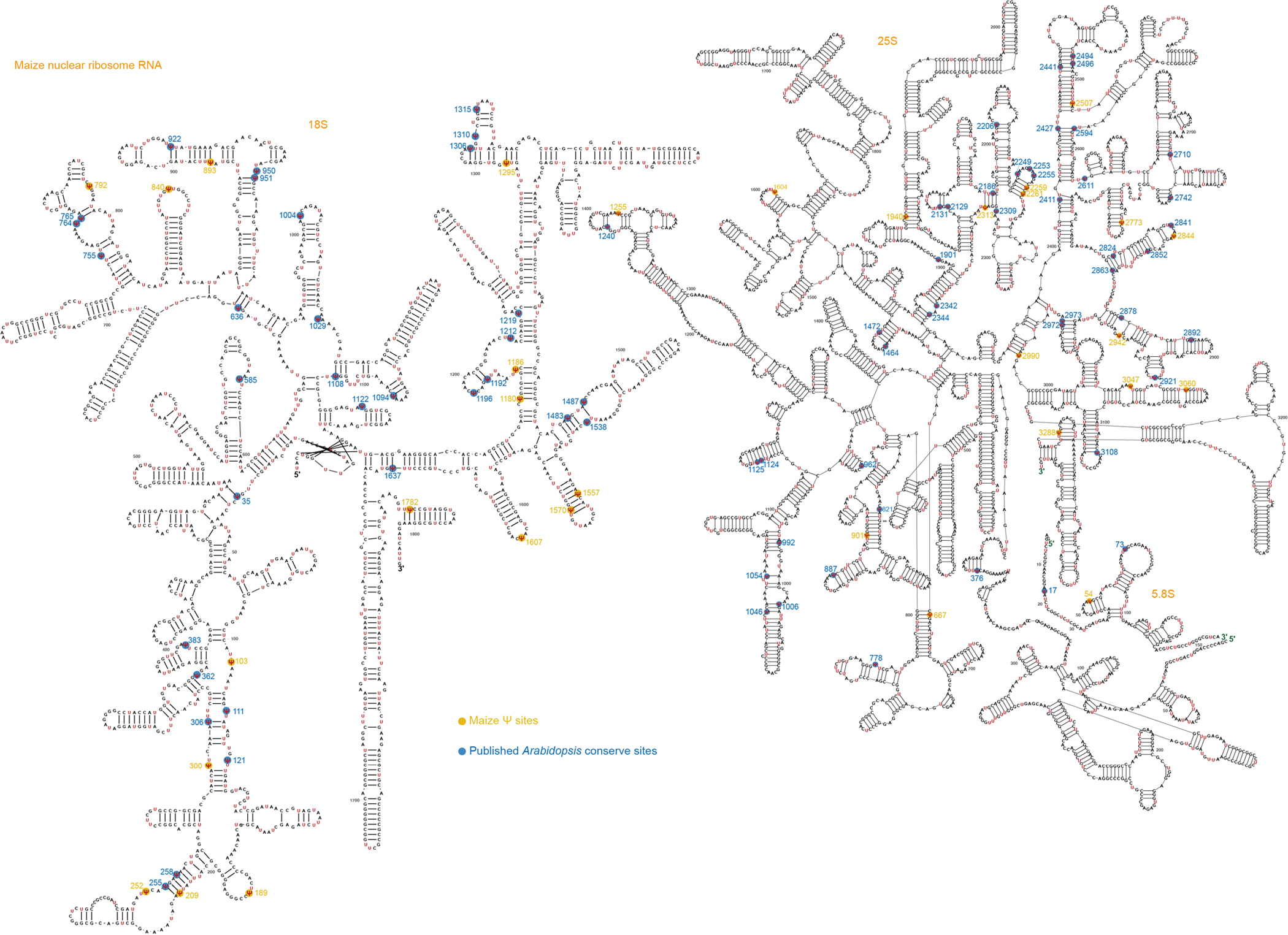
The conserved Ψ sites in the rRNA of nuclear 18S, nuclear 5.8S and nuclear 25S in maize and *Arabidopsis.* The Ψ sites were accurately marked and compared with previously published sites. Yellow color marks the Ψ sites only in maize. Blue color marks the Ψ sites conserved in both maize and *Arabidopsis*.

**Extended Data Fig. 4 |:**
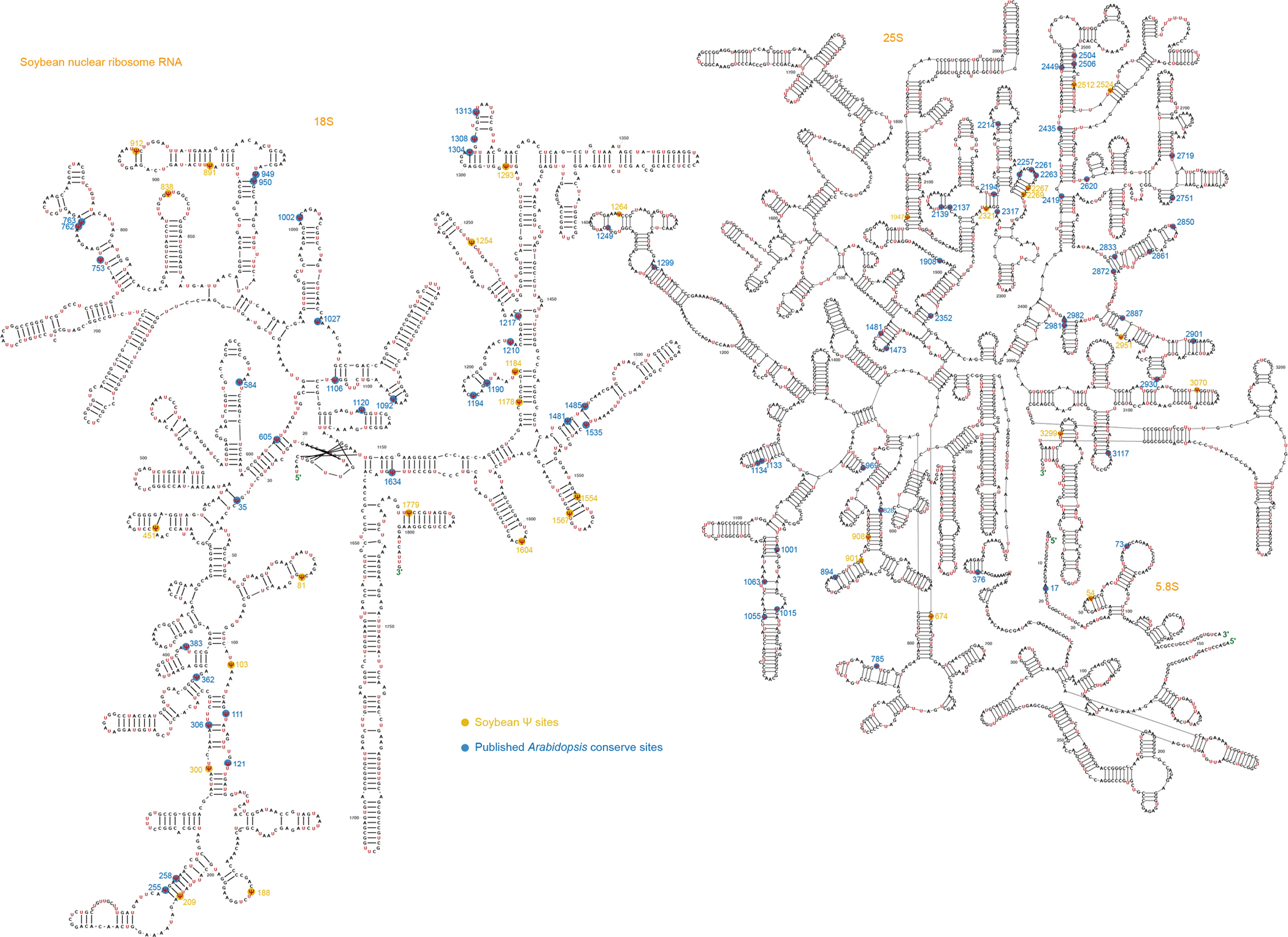
The conserved Ψ sites in the rRNAs of nuclear 18S, nuclear 5.8S and nuclear 25S in soybean and *Arabidopsis.* The Ψ sites were accurately marked and compared with previously published sites. Yellow color marks the Ψ sites only in soybean. Blue color marks the Ψ sites conserved in both soybean and *Arabidopsis*.

**Extended Data Fig. 5 |:**
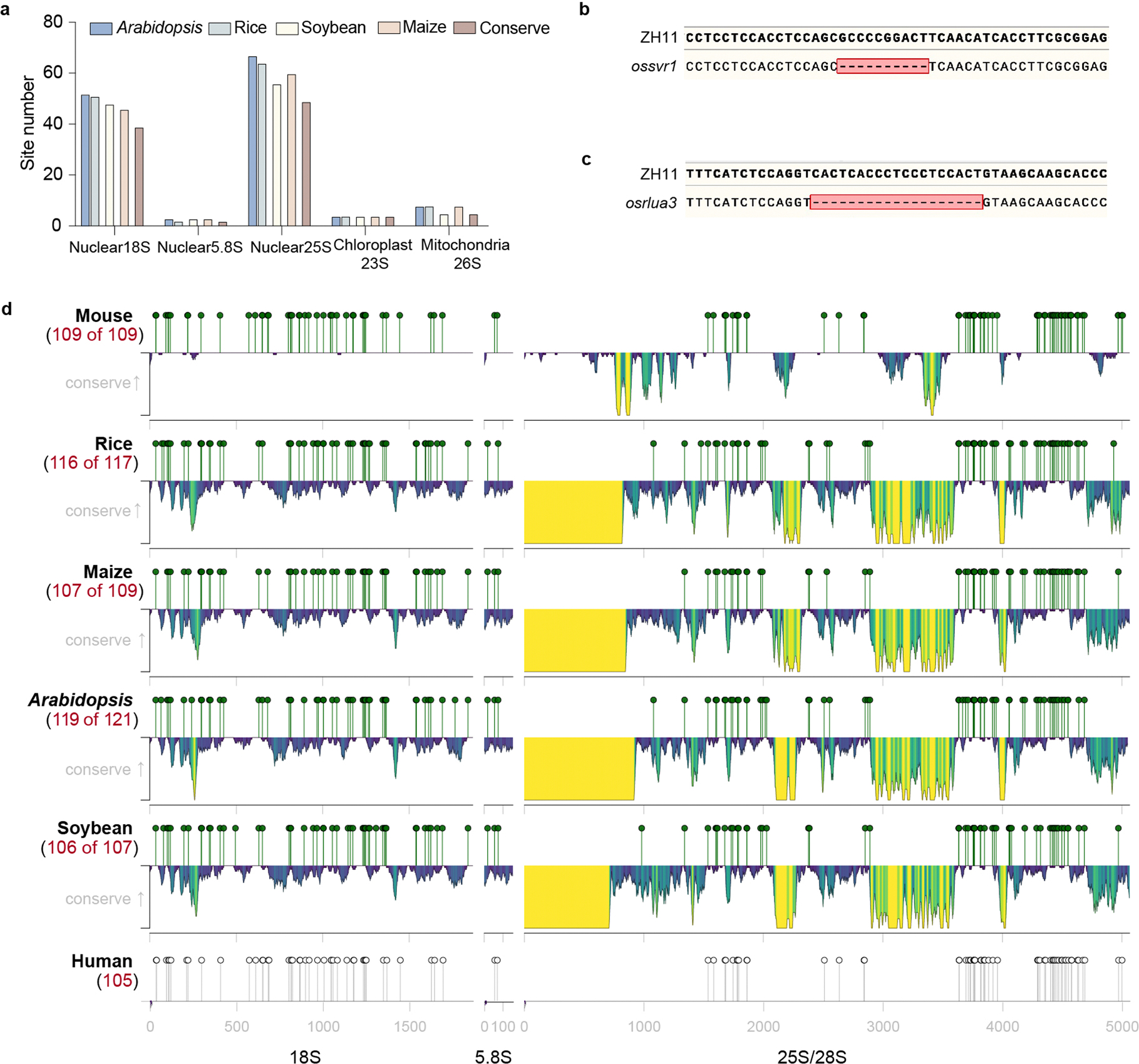
rRNA sequence conservation and rRNA Ψ sites among plant species and mammals. **a**, The number of total- and conserved Ψ sites in rRNAs of nuclear 18S, nuclear 5.8S, nuclear 25S, chloroplast 23S, and mitochondria 26S identified in the four plants. **b**, **c**, The mutation types of the *ossvr1* and *osrlua3* mutant lines, in the background of ZH11 using CRISPR-Cas9. **d**, Diagram comparing the rRNA sequence conservation and Ψ sites for nuclear encoded 18S, 5.8S and 25S/28S rRNAs in four plant species as well as mouse and human. The yellow to purple colors represent the rRNA sequence conservation scores ranging from low to high. Each spot represents a rRNA Ψ site in different species. The Ψ site numbers are labeled along with the species names. Number in red color stands for the number of Ψ sites aligned to human rRNA among the total number of nuclear encoded rRNA Ψ sites identified in corresponding species. A total of 105 Ψ sites are identified in nuclear encoded rRNA in human. The BID-seq datasets of mouse and human were downloaded from the Gene Expression Omnibus database under the accession number of GSE238245 and GSE179798 respectively.

**Fig. 1 |:**
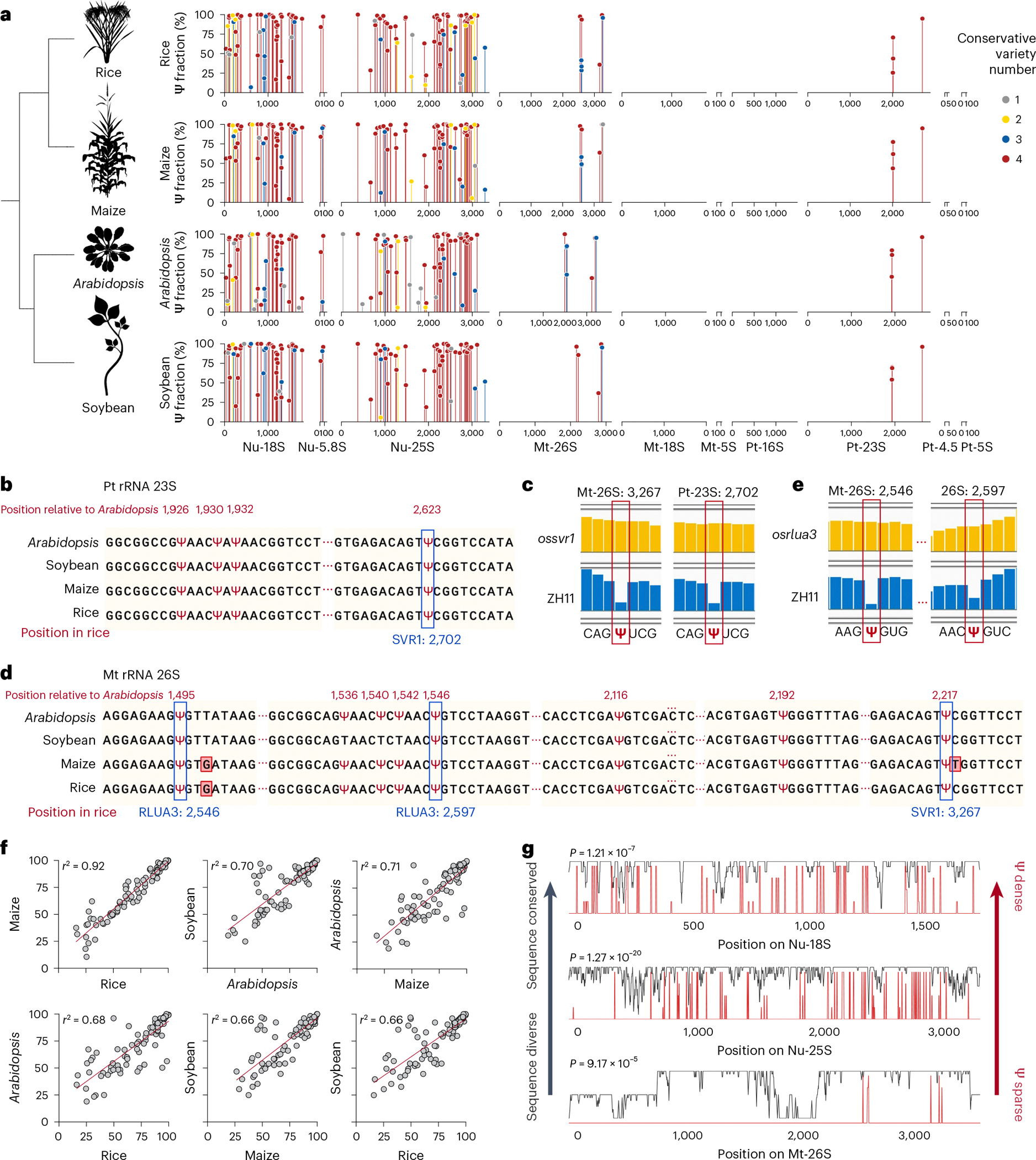
BID-seq reveals evolutionarily conserved pseudouridylation in rRNA across monocots and dicots. **a**, Exact positions and stoichiometry of Ψ sites in all rRNA genes across multiple plant species. Sites identified on nuclear-encoded rRNAs (Nu-18S, Nu-5.8S, Nu-25S), mitochondria-encoded rRNAs (Mt-26S, Mt-18S, Mt-5S) and chloroplast-encoded rRNAs (Pt-16S, Pt-23S, Pt-4.5S, Pt-5S). Grey dots denote Ψ sites on rRNA identified in only one species; yellow dots denote Ψ sites on rRNA conserved in two species; blue dots denote Ψ sites on rRNA conserved in three species; red dots denote Ψ sites on rRNA conserved in four species. **b**, Locations of the conserved rRNA Ψ sites in chloroplast-encoded 23S rRNA, with the Ψ sites deposited by OsSVR1 shown in the blue box. **c**, Integrative Genomics Viewer examples showing the OsSVR1-dependent Ψ sites in the Mt-26S rRNA and Pt-23S rRNA in rice, with the Ψ sites deposited by OsSVR1 shown in the red box. **d**, Location of the conserved rRNA Ψ sites in mitochondria 26S rRNA. The Ψ sites deposited by OsRLUA3 and OsSVR1 are shown in the blue box. **e**, Integrative Genomics Viewer examples showing the two OsRLUA3-dependent Ψ sites in the Mt-26S rRNA in rice, with the Ψ sites deposited by OsRLUA3 shown in the red box. **f**, Correlation of the Ψ fraction in rRNA between any two of the studied plant species. Grey dots denoted the Ψ sites in rRNA. Pearson’s *r* values are given above each panel. **g**, Diagram showing the rRNA sequence conservation (black line) for Nu-18S, Nu-25S and Mt-26S. Conservation scores were computed based on the similarity across the multiple sequence alignment of rRNA sequences from four species. Scores were scaled according to the maximum and minimum values observed. The red line shows that densely Ψ-modified regions correlate with more conserved rRNA sequences, whereas sparsely Ψ-modified regions correlate with the more diverse rRNA sequences. The *P* value was calculated by two-sided Pearson correlation test.

rRNA typically accounts for 90% of the total cellular RNA and is heavily pseudouridylated. In humans, approximately 8.8% of the U sites in rRNA are Ψ (104 of 1,191)^30^, whereas in yeast, this figure is about 3.8% (54 of 1,423)^31^. rRNA peudouridylation is less explored in plants with the modification stoichiometry of most pseudouridine sites largely unknown. To investigate this, we applied BID-seq for high-resolution mapping of Ψ in representative monocot and dicot plants—rice, maize, *Arabidopsis* and soybean (Fig. 1a)—using shoot samples. This study provides systematic mapping of the exact positions and stoichiometry of Ψ sites in all rRNAs across multiple plant species (Fig. 1a and Extended Data Figs. 1–4). This analysis encompasses rRNAs encoded in the nucleus (Nu-18S, Nu-5.8S, Nu-25S), mitochondria (Mt-26S, Mt-18S, Mt-5S) and chloroplast (Pt-16S, Pt-23S, Pt-4.5S, Pt-5S). Ψ modifications are found in all three subunits of rRNA encoded in the nucleus, whereas for rRNA encoded in the mitochondria and chloroplast, they are identified only in the large subunits, namely Mt-26S and Pt-23S (Fig. 1a). We observed Ψ modification sites in rRNA across four species: rice (129 sites), maize (121 sites), *Arabidopsis* (134 sites) and soybean (116 sites) (Extended Data Fig. 5a). Specifically, the numbers of Ψ sites in nucleus-encoded rRNA are 117, 109, 122 and 107 for rice, maize, *Arabidopsis* and soybean, respectively, accounting for approximately 10.9%, 10.1%, 10.2% and 9% of U sites as Ψ in the above four plant species. Notably, Mt-18S, Mt-5S, Pt-16S, Pt-4.5S and Pt-5S rRNAs do not have high-frequency Ψ modifications (Fig. 1a). In these plant species, rRNAs encoded in the nucleus show greater enrichment of Ψ sites compared with rRNAs encoded in the mitochondria and chloroplast (Fig. 1a).

The availability of enzymes to install Ψ might be key to its varied distribution in different rRNA species, because Ψ deposition on rRNAs encoded in the nucleus is mostly guided by small nucleolar RNA, whereas Ψ sites in mitochondria- and chloroplast-encoded rRNAs are catalysed by specific enzymes. For instance, SVR1 is known to install Ψ on chloroplast-encoded 23S rRNAs at position 2,702 (Ψ^2,702^) in rice, which corresponds to Ψ^2,623^ in *Arabidopsis*^15^ and Ψ^2,700^ in *Zea mays*^32^. Our data confirmed the presence of Ψ at this site in all three plant species, and also detected the same modification on homologue sites in soybean (Fig. 1b). To validate the effect of SVR1 on site Pt-23S:Ψ^2,702^, we performed BID-seq on a rice *svr1* mutant (Extended Data Fig. 5b) and found that the modification is quantitatively eliminated upon gene deletion (Fig. 1c). Furthermore, we discovered that SVR1 could deposit Ψ on mitochondria-encoded 26S rRNA in rice, which corresponds to Ψ^2,217^ in *Arabidopsis*, and this site is conserved among the four plant species studied (Fig. 1d). Another protein, OsRLUA3, is predicted to convert U to Ψ in rice. BID-seq data analysis on a rice *osrlua3* mutant (Extended Data Fig. 5c) revealed that the two Os*rlua3*-dependent Ψ sites on mitochondria-encoded 26S rRNA (Fig. 1d,e) were also identified in the other three plant species, indicating the conserved role of OsRLUA3 on pseudouridylation of mitochondria-encoded 26S rRNA in the plant kingdom.

A notable proportion of rRNA Ψ sites are conserved across the four plant species. This includes 39, 2 and 49 conserved sites in the nuclear-encoded 18S, 5.8S and 25S rRNAs, respectively (Extended Data Fig. 5a). In addition, four conserved Ψ sites in the chloroplast-encoded 23S rRNA and eight in the mitochondria-encoded 26S rRNA were identified in these plant species (Fig. 1b–e). Interestingly, the Ψ fraction of the conserved rRNA sites between any two of the four plants species is well correlated (*r* = 0.66–0.92) (Fig. 1f). Our comparison of conservation scores between genomic sequences and Ψ profiles reveals a higher density of Ψ modifications in sequence conserved regions than in variable regions (Fig. 1g). This suggests conserved roles for rRNA Ψ modifications in maintenance of the integrity and functionality of rRNA in most plant species. More interestingly, Ψ modifications in sequence conserved regions are also more prevalent than variable regions both in human^27^ and mouse^15^ (Extended Data Fig. 5d). This suggests conserved roles for rRNA Ψ modifications in the overall rRNA structure or function in most plant species, as well as mammals.

### Ψ stoichiometries on rRNA and tRNA regulate translation

**Fig. 2 |:**
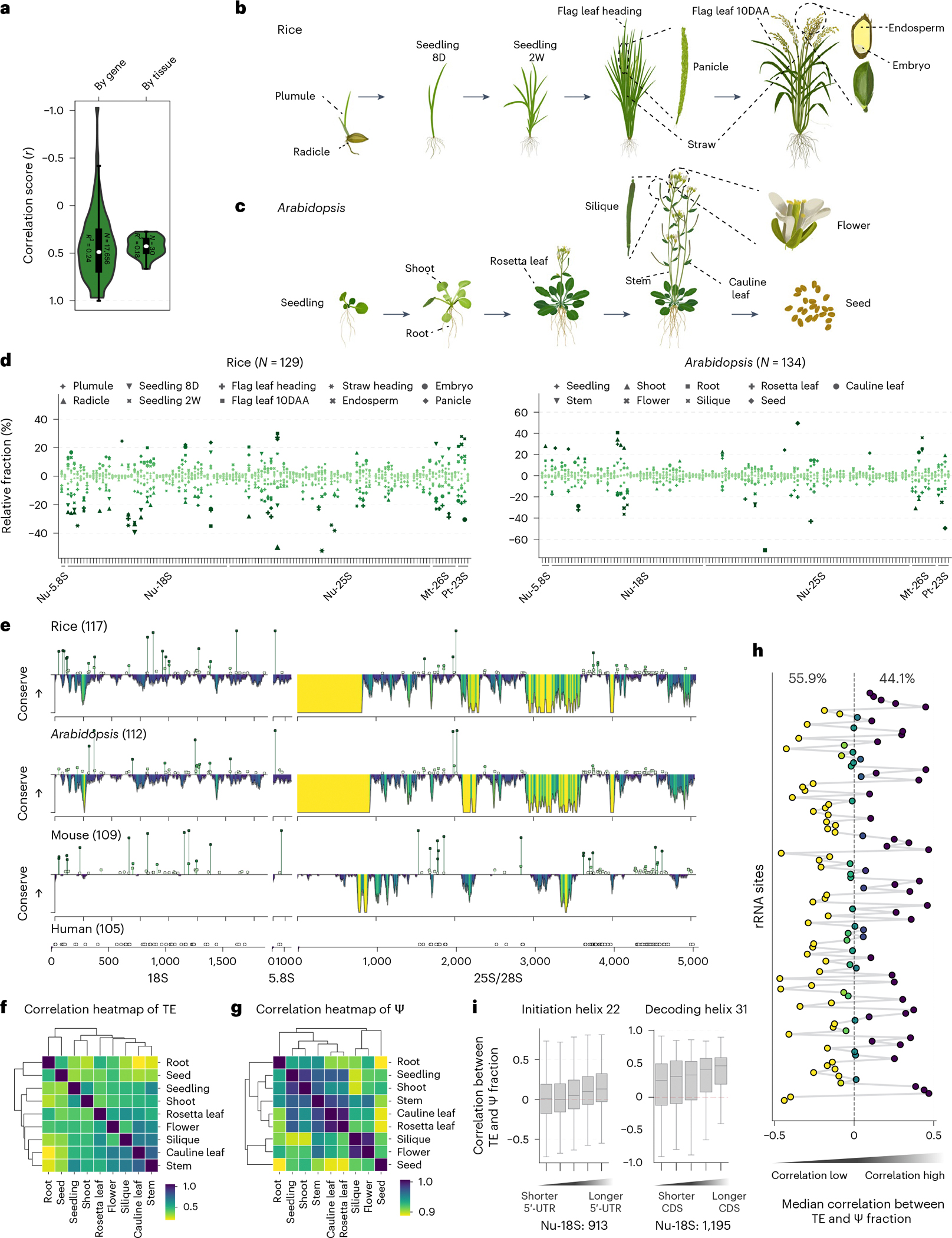
Ψ stoichiometries on rRNA modification sites affect translation across different tissues. **a**, The correlation between mRNA level and protein abundance in *Arabidopsis* is quantified at both the tissue level (determined by analysing all genes identified in *Arabidopsis*) and gene level (assessed across all tissue samples associated with a single gene in *Arabidopsis*). Proteome and RNA expression level data were downloaded from PXD013868 (ref. 33). **b**,**c**, Ten rice (**b**) and nine *Arabidopsis* (**c**) organs were subjected to BID-seq. **d**, Relative fraction of rRNA Ψ sites across different tissues in rice and *Arabidopsis*. **e**, Diagram comparing the nuclear rRNA sequence conservation with individual rRNA Ψ sites in rice, *Arabidopsis*, mouse and human. The yellow to purple colours represent the sequence conservation scores ranging from low to high. Each spot represents a rRNA Ψ site and spot height represents modification variations. The Ψ site number is labelled along with the species name. The BID-seq datasets of mouse and human were downloaded from GSE238245 and GSE179798, respectively. **f**, Correlation heatmap showing the translation efficiency (TE) of each *E*_transcript_ among *Arabidopsis* tissues. *E*_transcript_ was calculated by normalizing ribosome-bound RNA to the mRNA (TPM/TPM). **g**, Correlation heatmap showing distinct rRNA Ψ stoichiometry patterns observed in different *Arabidopsis* tissues. **h**, Correlation between modification fraction of all ribosomal Ψ sites with *E*_transcript_. The median correlation score of each site is shown. Yellow to purple dots represent the correlation score from small to large. **i**, Correlation between modification fraction of all ribosomal Ψ sites with *E*_transcript_ of different gene groups classified by relative length of 5′-UTR and CDS. A total of 977 genes were divided into five equal-sized groups based on their quantiles. Relative length of the 5′-UTR was defined as the value of the 5′-UTR length compared with its CDS length. The line shows the median, boxes represent the interquartile range (IQR) and whiskers extend to 1.5× IQR. 10DAA, 10 days after anthesis; 8D, 8 days; 2W, 2 weeks.

The flow of genetic information from the transcriptome to the proteome is complex, and RNA expression levels do not always align closely with protein abundance. For instance, in *Arabidopsis*, proteome data from 30 tissues indicate that RNA levels can explain only 18% of the variation in protein abundance in a single sample^33^ (Fig. 2a). Codon usage, protein interactions, UTR motifs and other genetic factors account for an additional 31% of the variation^33^. However, a notable proportion (48%) of the variance in protein abundance remains unexplained^33^. Furthermore, when considering the expression of a single gene across several tissues—in which genetic factors are constant—the RNA level still accounts for only 24% of the variance in protein abundance^33^ (Fig. 2a). This discrepancy suggests that epitranscriptomic factors, like Ψ modification, might contribute substantially to protein abundance regulation. To investigate this, we mapped Ψ sites and their stoichiometry in ten rice tissues (plumule, radicle, seedling at 8 days, seedling at 2 weeks, straw at heading, flag leaf at heading, panicle, flag leaf at 10 days after anthesis, embryo at 10 days after anthesis and endosperm at 10 days after anthesis) and nine *Arabidopsis* tissues (seedling, shoot, root, rosetta leaf, cauline leaf, stem, flower, silique and seed) (Fig. 2b,c). We compared ribosomal Ψ sites across different tissues in rice (129 sites) and *Arabidopsis* (134 sites) (Fig. 2d), with marked variations observed across different tissues in certain regions (Fig. 2d). Similar to rice and *Arabidopsis*, the modification variations across tissues are also notable in mouse^15^, especially for the conserved genomic sequence on the 25S/28S rRNA in both plants and mammals (Fig. 2e). This probably suggests that tissue-dependent Ψ modification modulates tissue-selective translation of the corresponding transcriptome.

**Extended Data Fig. 6 |:**
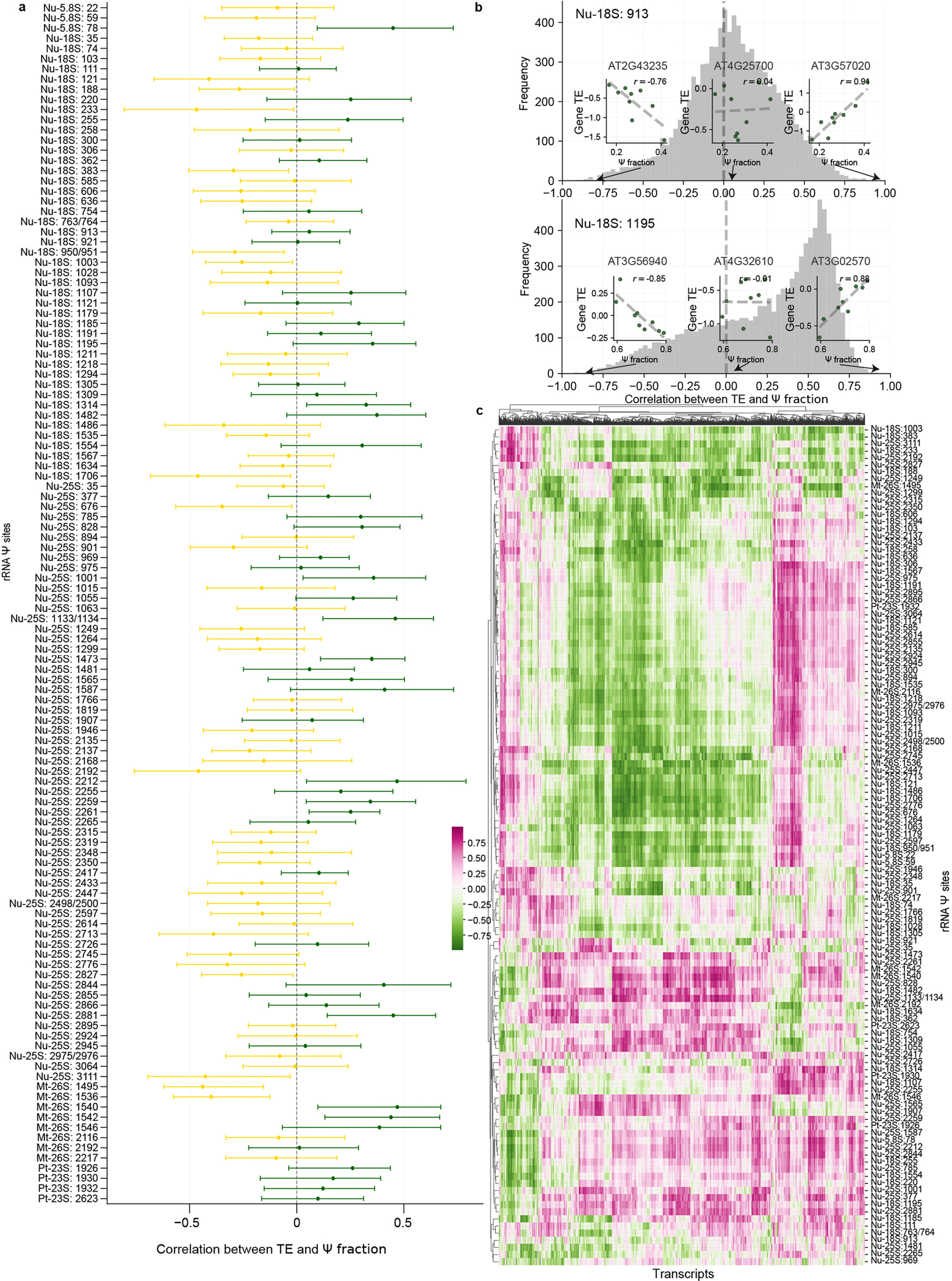
Correlation analysis of Ψ level with translation efficiency of genes across multiple tissues in *Arabidopsis.* **a**, Correlation analysis between Ψ level on each rRNA site and 977 transcripts’ translation efficiency at transcript level (E_transcript_). The median correlation value was shown by the yellow or the blue dot. The error bars represent the interquartile range (IQR). E_transcript_ were calculated by normaling ribosome-bound RNA (with polysome footprinting data) to the whole-cell mRNA level. **b**, Examples of Ψ sites on Nu-18S: 913 and Nu-18S: 1195 that calculated with the correlations between Ψ levels and translation efficiency (E_transcript_) among nine *Arabidopsis* tissues are shown. E_transcript_ were calculated by normaling ribosome-bound RNA (with polysome footprinting data) to the whole-cell mRNA level. Examples of correlations regarding to specific genes were also showed in **b**. Nu-18S: 913 belongs to the initiation region, and Nu-18S: 1195 belongs to the decoding region. **c**, Heatmap showing all the correlation value between the Ψ level of each rRNA site and the translation efficiency (E_transcript_) of each transcript. The correlation matrix was clustered by the similarities score among Ψ sites and 977 transcripts.

To assess the impact of translational regulation on translation efficiency at the transcript level (*E*_transcript_), we performed polysome profiling sequencing with the nine *Arabidopsis* tissues and calculated the translation efficiency for each gene by normalizing the ribosome-bound RNA level to the whole-cell transcript level. Our analysis revealed that root and seed tissues, which are highly specialized in their development, have marked differences in translation efficiency compared with other tissues (Fig. 2f). Similarly, distinct rRNA Ψ stoichiometry patterns were observed in these tissues, as revealed by our BID-seq datasets (Fig. 2g). We next calculated the correlation between the Ψ modification fraction of all ribosomal Ψ sites and the translation efficiency (*E*_transcript_) of different genes (Extended Data Fig. 6a,b). At the overall level, based on the median correlation score of Ψ sites on rRNA with all transcripts, 55.9% of sites showed a negative correlation and 44.1% showed a positive correlation (Fig. 2h). This suggests that Ψ modifications at different regions of rRNA influence translation efficiency in varying ways. Transcripts associated with Ψ sites showing a positive or negative correlation had distinct clustering patterns, suggesting that Ψ levels on rRNA may influence translation efficiency in a transcript-specific manner (Extended Data Fig. 6c).

Hypo-pseudouridylation of rRNA in the *cbf5* mutant yeast, and for its homologue *dkc1* in mouse and human, could lead to decreased translational fidelity and a reduction in internal ribosome entry site-dependent translational initiation^19–22^. In addition, mRNAs with short 5′-UTRs have lower-than-average translational efficiency in yeast^34^. We then explored the correlation between rRNA Ψ fraction and translation efficiency for different groups of transcripts in different *Arabidopsis* tissues using rRNA Ψ sites of Nu-18S:913 (initiation region) and Nu-18S:1,195 (decoding region) as examples, respectively. We found that gene transcripts with a longer 5′-UTR show more positive correlation between Ψ level and translation efficiency for Ψ sites at rRNA initiation regions (Fig. 2i), whereas gene transcripts with greater CDS length show more positive correlation between Ψ level and translation efficiency for Ψ sites at rRNA decoding regions (Fig. 2i).

**Fig. 3 |:**
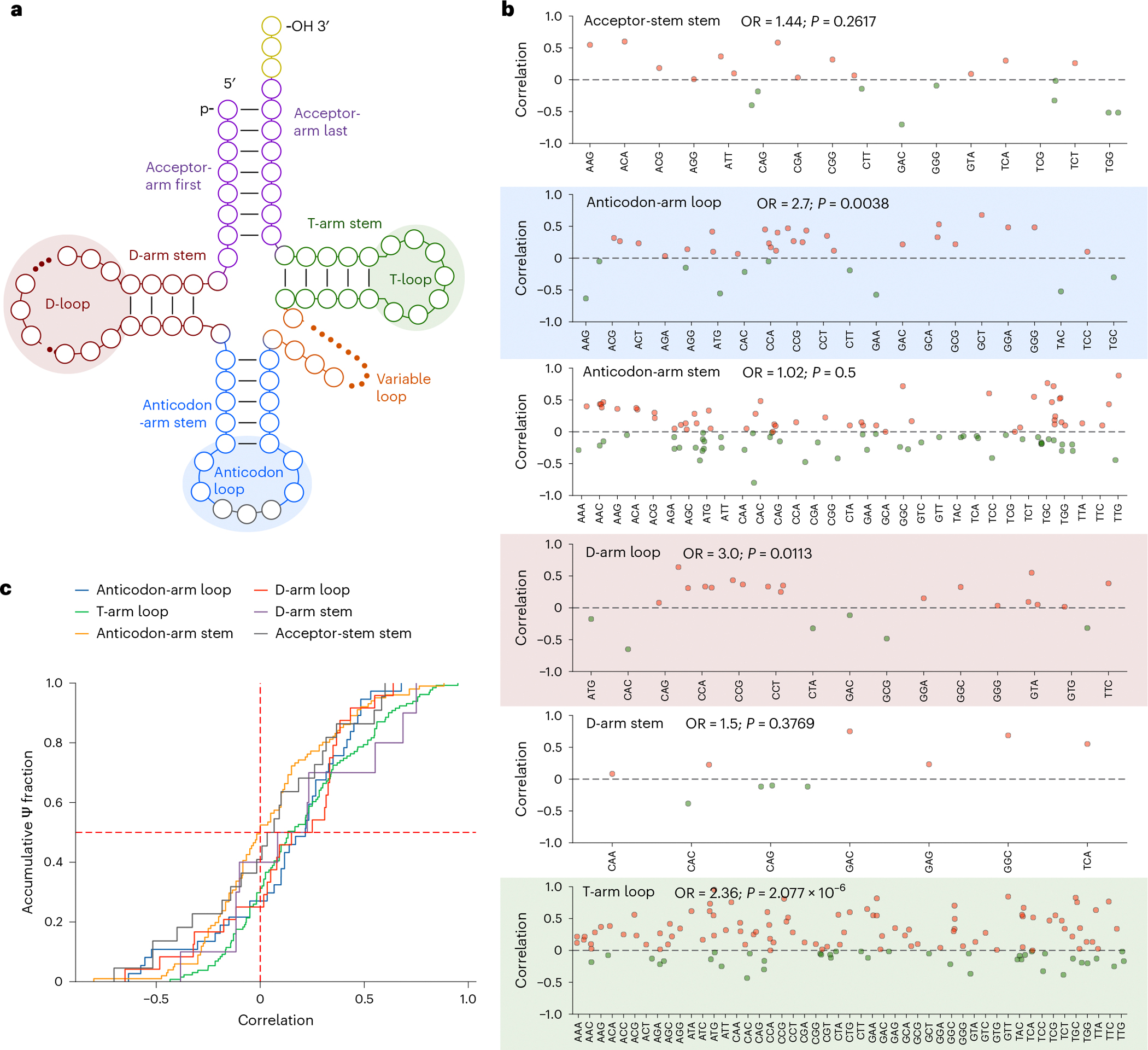
Ψ stoichiometries on tRNA affect translation across different plant tissues. **a**, tRNA two-dimensional structures. The loop and stem regions are shown. **b**, Correlations between Ψ stoichiometry in different tRNA regions and the translation efficiency of their respective codons. Ψ stoichiometry in different regions of tRNA in all the *Arabidopsis* tissues was used to calculate the correlation. Translation efficiency was calculated from the density of ribosomes bound to each mRNA molecule in the entire polysome profiling dataset with the codon frequency in the mRNA of the whole transcriptome. The labelled odds ratio (OR) was calculated from the number of positives (red) divided by the number of negatives (green). The *P* value was determined by one-tailed binomial test with a null hypothesis of 0.5. **c**, Accumulative fraction of the correlation between Ψ stoichiometry in different tRNA regions and the translation efficiency of their respective codons.

tRNAs, as the most abundant molecules in the cell, are also enriched with high-stoichiometry Ψ sites. Previous research has suggested that modifications in tRNA can influence decoding speed^35–39^ and affect translation by fine-tuning tRNA structures and their interactions with the corresponding mRNA codons^40^. To explore whether Ψ stoichiometry in tRNA regulates translation efficiency across different tissues, we first computed the translation efficiency at the codon level (*E*_codon_) using polysome profiling data from different tissues of *Arabidopsis*. Codon translation efficiency was calculated using ribosome footprinting data (Methods). We then re-analysed the total RNA BID-seq datasets that originated from all the *Arabidopsis* tissues. In total, we detected 144 tRNAs covering 51 anticodons and computed Ψ sites with stoichiometry in each identified tRNA (Supplementary Table 1). We observed strong positive correlations between Ψ stoichiometry in the T-arm loop of different tRNAs and the translation efficiency of their respective codons (Fig. 3a,b). This is consistent with a previous report that Ψ modifications in the T-arm loop of tRNA globally control translation in *Escherichia coli*^41^. Similarly, significantly positive correlations were observed for Ψ sites in both the anticodon-arm loop and D-arm loop, although these were not as strong as that of the T-arm loop in *Arabidopsis* (Fig. 3b). By contrast, correlations between Ψ stoichiometry in the acceptor-stem stem, anticodon-arm stem or D-arm stem and the translation efficiency of their respective codons were more evenly dispersed than the loop regions, which were not significantly correlated in our analysis (Fig. 3b,c). These results indicate that tRNA loop regions are potentially more critical for the regulation of protein biosynthesis or translation than the stem regions in *Arabidopsis*.

### Limited interspecies conservation for plant mRNA Ψ sites

**Extended Data Fig. 7 |:**
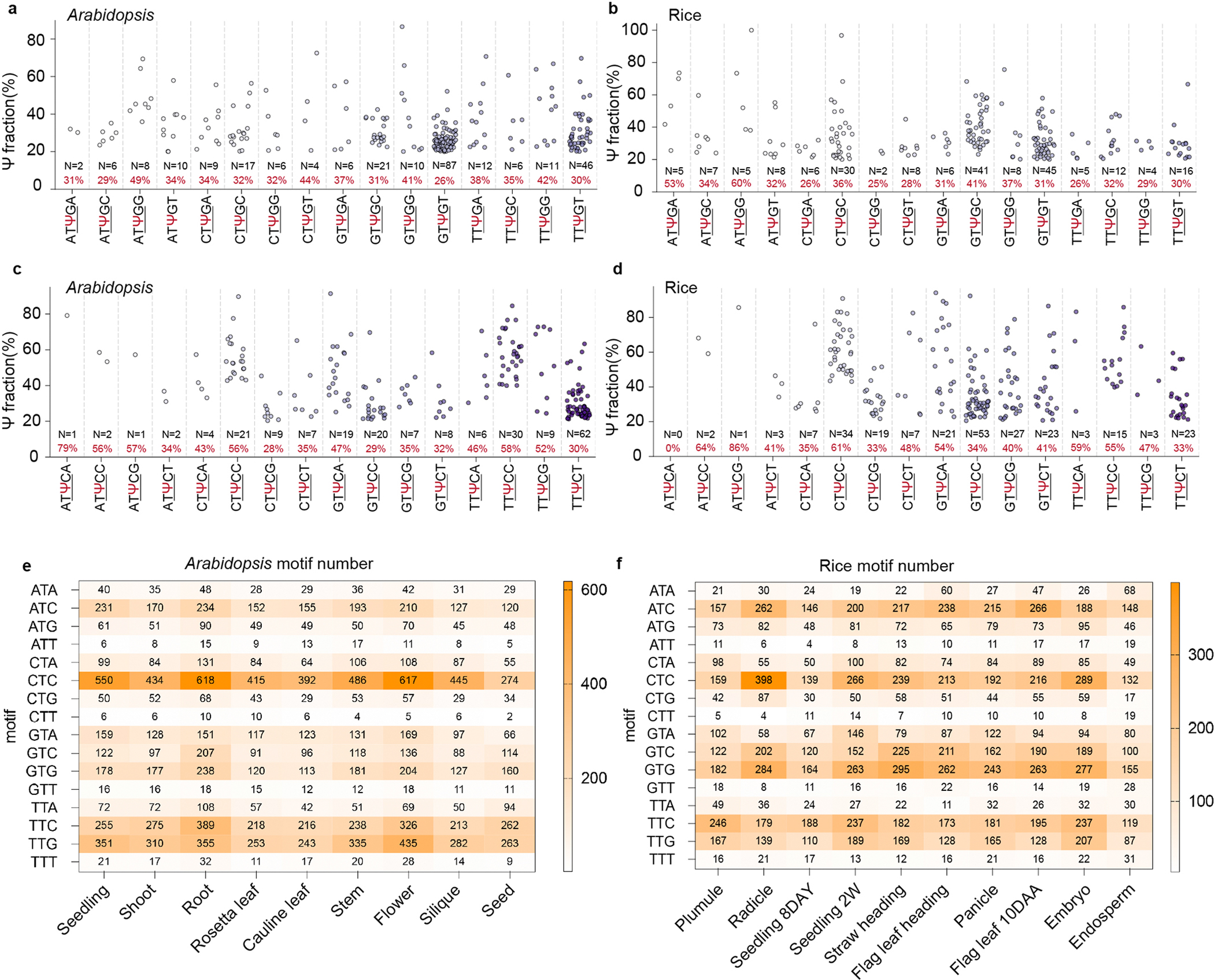
Ψ modification motifs in *Arabidopsis* and rice. **a**, **b**, All potential T**Ψ**G motif sequences with a sliding window of 1nt in *Arabidopsis* (**a**) and rice (**b**). Motifs with fractions over 20% were selected to plot the figure and the number of each motif (N) is shown. **c**, **d**, All potential T**Ψ**C motif sequences with a sliding window of 1nt in *Arabidopsis* (**c**) and rice (**d**). All the detected Ψ sites within tissues were combined to calculate the motifs. Motifs with fractions over 20% were selected to plot the figure and the number of each motif (N) is showed. The sliding window of 1 nt means 1 nucleotide upstream and 1 nucleotide downstream of the given motif. Combining with the motif length of 3 nt, the window size is 5 nt. **e**, **f**, Heatmap showing motif types and numbers of Ψ modifications in each tissue of *Arabidopsis* (**e**) and rice (**f**). All the detected Ψ sites within tissues were combined to calculate the motifs. 8DAY represents 8 days and 2 W represents 2 weeks. 10DAA represents 10 days after anthesis.

**Extended Data Fig. 8 |:**
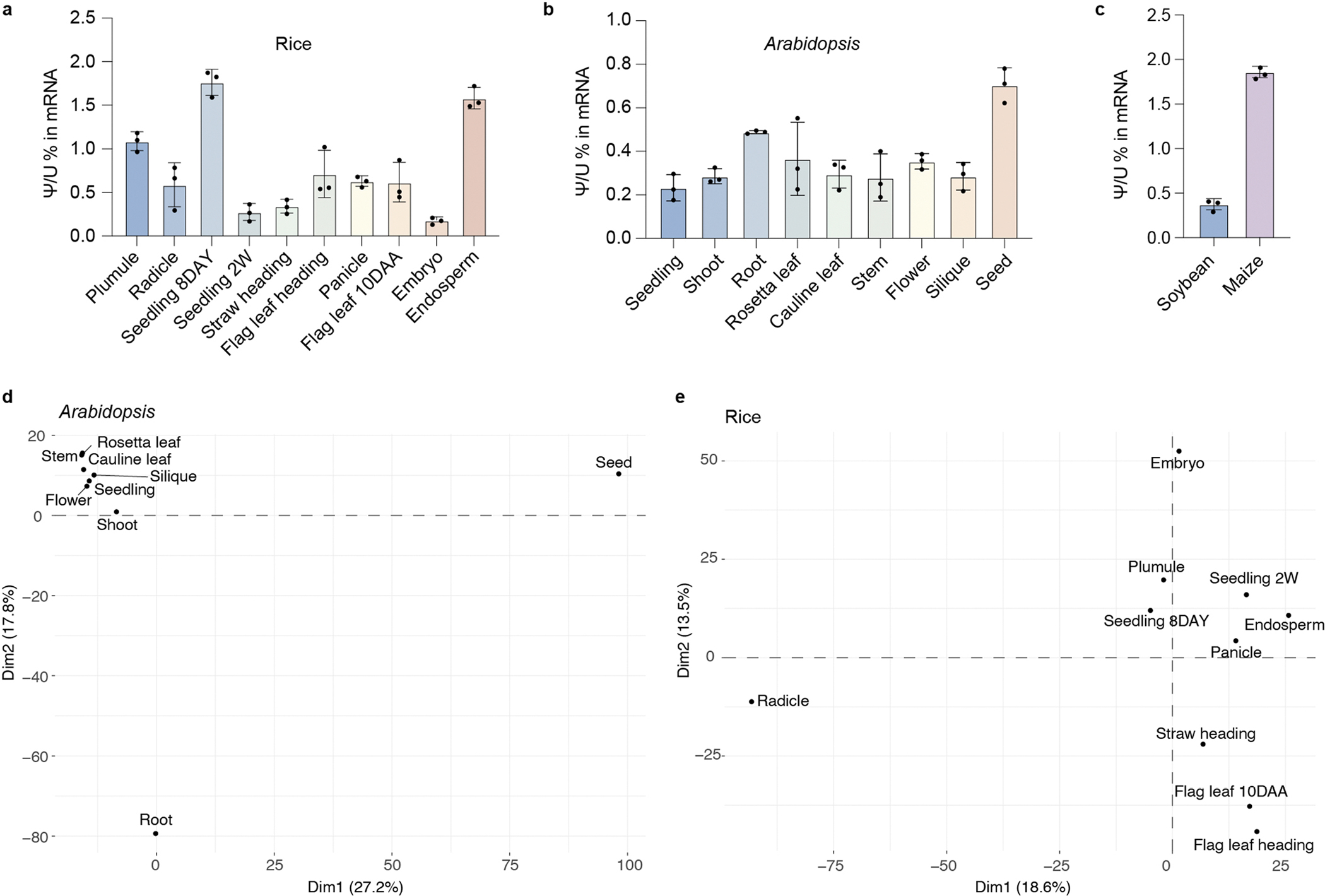
Overview of the quality of BID-seq data. **a**–**c**, mRNA Ψ levels in the harvested samples were quantified using LC-MS/MS for rice (**a**), *Arabidopsis* (**b**), maize and soybean (**c**). Three biological replicates were used. Data are means ± SD, *n* = 3. **d**, **e**, Principal component analysis (PCA) of Ψ fractions in *Arabidopsis* (**d**) and in rice (**e**) across different tissues. 8DAY represents 8 days and 2 W represents 2 weeks. 10DAA represents 10 days after anthesis.

**Fig. 4 |:**
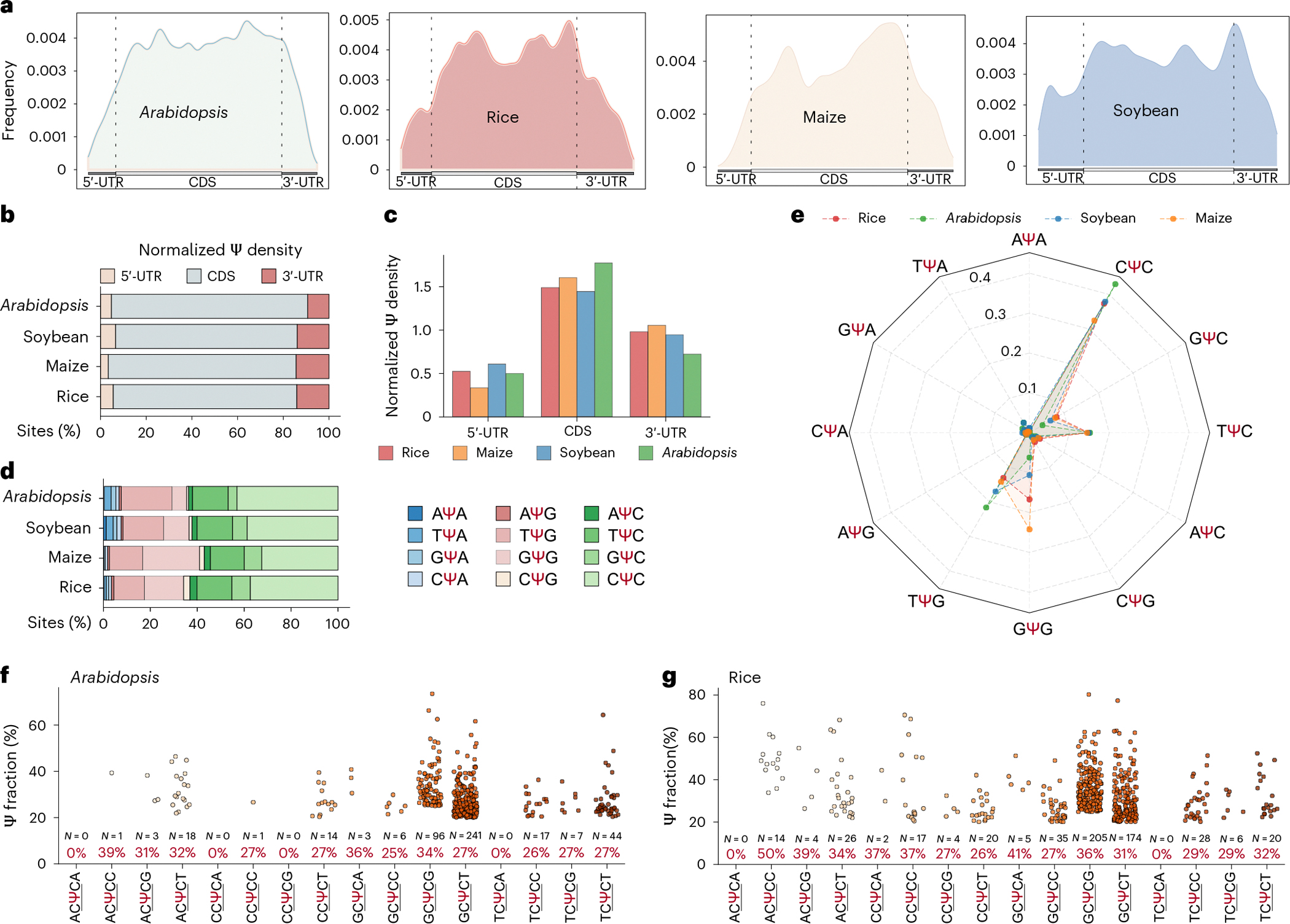
BID-seq identifies abundant mRNA Ψ sites across different plant species. **a**, Metagene profiles showing Ψ site distribution across transcripts in four plant species. Each transcript is segmented into three regions: 5′-UTR, CDS and 3′-UTR. We combined Ψ sites from all rice and *Arabidopsis* tissues to plot metagene profiles. **b**, Site percentage of normalized Ψ density distributed in 5′-UTR, CDS and 3′-UTR in the four plant species. **c**, Normalized Ψ density across 5′-UTR, CDS and 3′-UTR in the four plant species. **d**, Deposition motif types of the four plant species. The site percentages of each motif are shown. **e**, Average fractions of the enriched motifs. **f**,**g**, Five-nucleotide motifs in *Arabidopsis* (**f**) and rice (**g**) containing CΨC sequences. The sliding window of one nucleotide means one nucleotide upstream and one nucleotide downstream of the given motif (CΨC). Combined with the motif length of three nucleotides (underlined), the window size is five nucleotides. *N*, number of Ψ sites; %, average Ψ fractions.

We next investigated Ψ sites observed in the mRNA of monocots and dicots, which had not been possible previously. In total, we identified 2,521 Ψ sites in rice, 388 in maize, 1,347 in soybean and 2,566 in *Arabidopsis* in shoot mRNA samples. We investigated the evolutionary conservation of mRNA Ψ modification in the homologous genes between any two of the four species (Methods). Intriguingly, almost no conserved sites were identified in the orthologous gene pairs between any two of the four species, potentially implying that mRNA Ψ sites are generally not evolutionarily conserved in plants. We analysed the distribution of Ψ in the metagene profile, and found that most Ψ sites are highly enriched in CDS, followed by 3′-UTR and 5′-UTR in the four plant species (Fig. 4a). The Ψ density normalized by the number of total nucleotides to gene structure shows a similar distribution pattern to that observed in the metagene profile (Fig. 4b,c). We next analysed the types of Ψ modification motifs for mRNA Ψ sites in the four species. Plant shoots show the same motifs but different motif proportions (Fig. 4d). The most enriched motif is CΨC, followed by TΨG and TΨC in rice, soybean and *Arabidopsis*, whereas the GΨG motif is particularly enriched in maize compared with the other three species (Fig. 4d), which may suggest divergence between monocots and dicots (Fig. 4e). Noticeably, the most prevalent motif, CΨC, is a new modification motif and has not been reported previously. Viewing all deposition sequences of the CΨC (Fig. 4f,g), TΨG (Extended Data Fig. 7a,b) and TΨC (Extended Data Fig. 7c,d) motifs using a sliding window of one nucleotide (one nucleotide upstream and one nucleotide downstream of the given three-nucleotide motif), the motif types are largely conserved between rice and *Arabidopsis*, although with different abundances (Extended Data Fig. 8e,f).

### mRNA Ψ profile is highly diverse between tissues

The poly(A)-tailed RNA of each biological replicate was purified and subjected to liquid chromatography tandem mass spectrometry (LC–MS/MS) to measure the ratio of Ψ to U. The ratio of Ψ to U in poly(A)-tailed RNA from these different tissues varies from 0.31% to 1.89% in rice (Extended Data Fig. 8a) and from 0.21% to 0.79% in *Arabidopsis* (Extended Data Fig. 8b), whereas the ratio in shoot is rather high in soybean and maize (Extended Data Fig. 8c). BID-seq enables detection of abundant Ψ sites in various plant tissues of rice and *Arabidopsis* using poly(A)-tailed RNA (Extended Data Fig. 8d,e). We obtained 8,512 mRNA Ψ sites from 5,739 gene transcripts through combining all the sites detected from all *Arabidopsis* tissues in this study, with the number of Ψ sites among nine *Arabidopsis* tissues ranging from 6,640 in rosetta leaf to 7,406 in flower (Supplementary Table 2). In rice, a total of 9,126 Ψ sites were detected from 5,876 gene transcripts across tissues, whereas the number of Ψ sites from ten different rice tissues ranges from 6,463 in radicle to 7,212 in seedling 2W (Supplementary Table 3).

**Extended Data Fig. 9 |:**
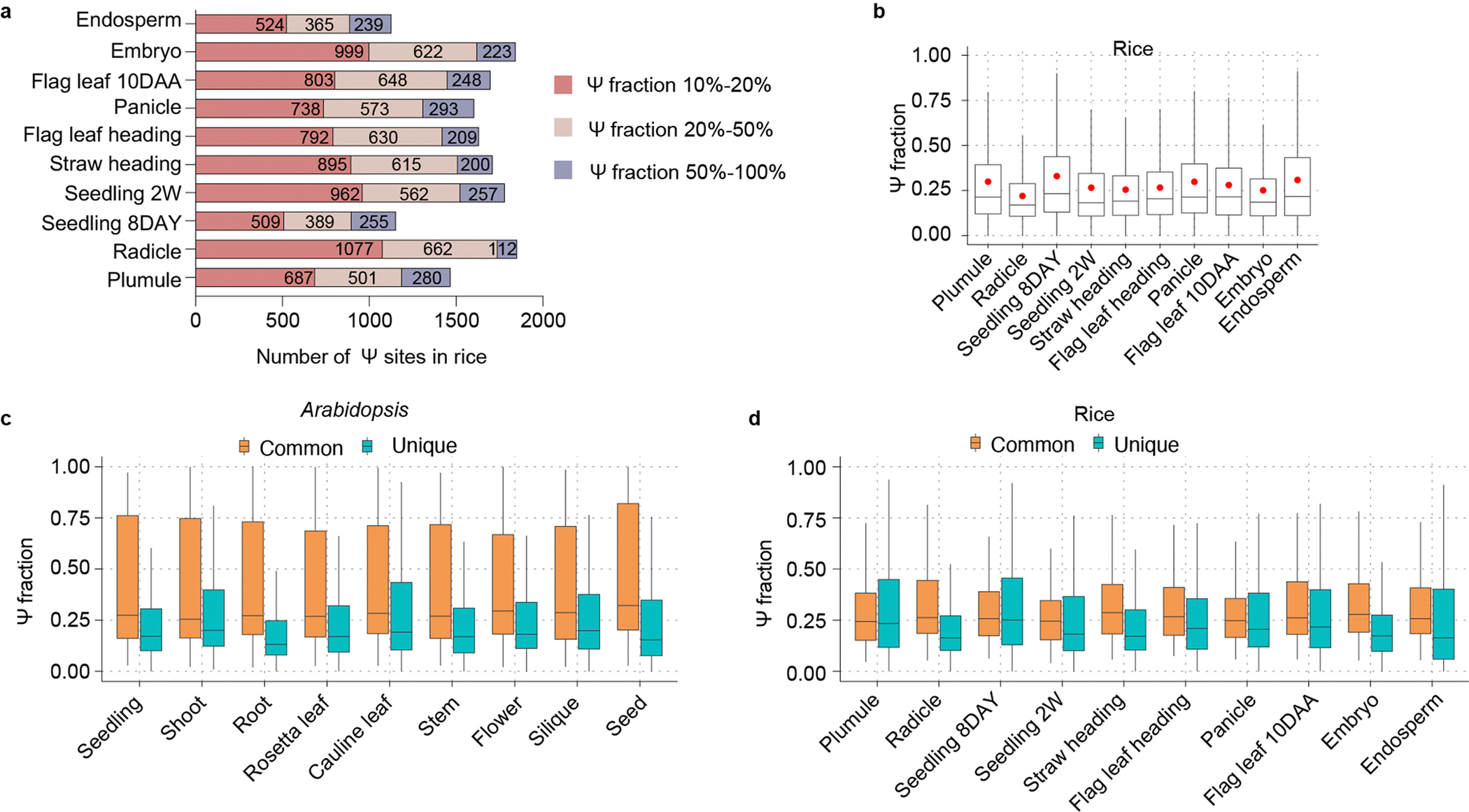
mRNA Ψ modification in various tissues of *Arabidopsis* and rice. **a**, BID-seq revealed a large number of mRNA Ψ sites in ten rice tissues. **b**, Diverse average Ψ fractions on mRNA across different tissues in rice, including plumule (*n* = 1,468), radicle (*n* = 1,851), seedling 8DAY (*n* = 1,153), seedling 2 W (*n* = 1,781), straw heading (*n* = 1,710), flag leaf heading (*n* = 1,631), panicle (*n* = 1,604), flag leaf 10DAA (*n* = 1,699), embryo (*n* = 1,844), and endosperm (*n* = 1,128) shown in boxplot. The red dots mark the mean, the lines show the median, the boxes represent the interquartile range (IQR), and the whiskers extend to 1.5× of the IQR. **c**, Boxplot showing fractions of tissue common (*n* = 123) and unique mRNA Ψ sites in *Arabidopsis*, including seedling (*n* = 478), shoot (*n* = 400), root (*n* = 1,160), rossta leaf (*n* = 385), cauline leaf (*n* = 315), stem (*n* = 416), flower (*n* = 706), silique (*n* = 370), and seed (*n* = 712). **d**, Boxplot showing fractions of tissue common (*n* = 55) and unique mRNA Ψ sites in rice, including plumule (*n* = 399), radicle (*n* = 1,042), seedling 8DAY (*n* = 200), seedling 2 W (*n* = 552), straw heading (*n* = 408), flag leaf heading (*n* = 509), panicle (*n* = 440), flag leaf 10DAA (*n* = 482), embryo (*n* = 728), and endosperm (*n* = 401). The lines show the median, the boxes represent the interquartile range (IQR), and the whiskers extend to 1.5× of the IQR. 8DAY represents 8 days and 2 W represents 2 weeks. 10DAA represents 10 days after anthesis.

**Fig. 5 |:**
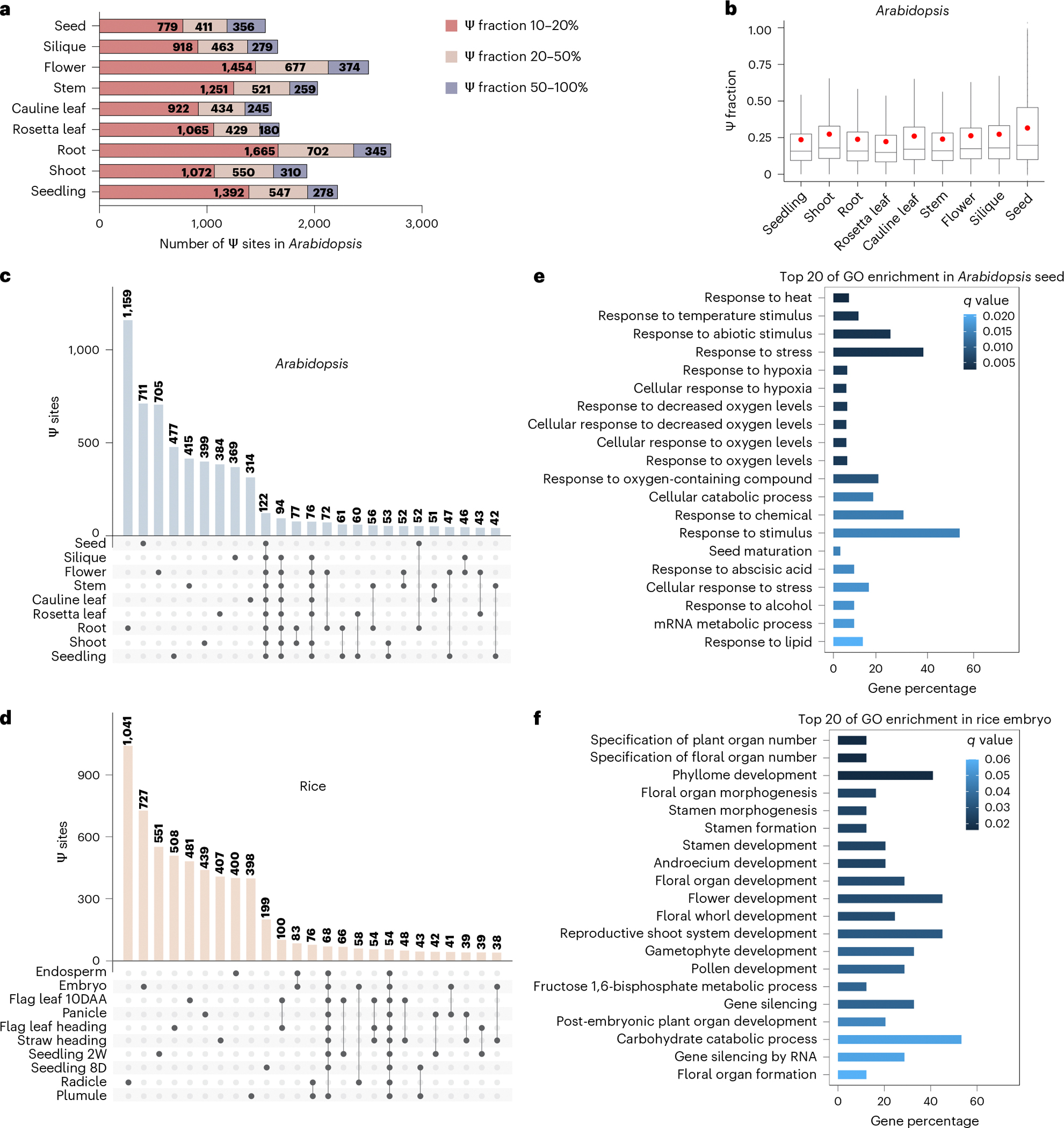
Comprehensive base-resolution maps of mRNA Ψ sites in rice and *Arabidopsis.* **a**, BID-seq revealed a large number of mRNA Ψ sites in nine *Arabidopsis* tissues. **b**, Ψ sites across nine *Arabidopsis* tissues, including seedling (*n* = 2,217), shoot (*n* = 1,932), root (*n* = 2,712), rosetta leaf (*n* = 1,764), cauline leaf (*n* = 1,601), stem (*n* = 2,031), flower (*n* = 2,217), silique (*n* = 2,505) and seed (*n* = 1,546), are shown as boxplots. Red dots mark the mean, lines show the median, boxes represent the IQR and whiskers extend to 1.5× IQR. **c**,**d**, Bar plot showing the number of tissue-unique and tissue-shared Ψ sites in nine *Arabidopsis* tissues (**c**) and ten rice tissues (**d**). **e**, GO enrichment analysis of mRNA with seed-specific Ψ sites in *Arabidopsis*. **f**, GO enrichment analysis of mRNA modified with specific Ψ sites in rice embryo.

The stoichiometry information facilitates the calculation of Ψ deposition fractions^13^. We found that more than two-thirds of all Ψ sites have a low Ψ fraction (<20%), and far fewer Ψ sites are highly modified (Ψ fractions >50%) in different tissues of both rice (Extended Data Fig. 9a) and *Arabidopsis* (Fig. 5a). This differs from mammals, in which we observed a higher percentage of highly modified mRNA Ψ sites (Ψ fractions >50%)^8,29^. To rule out potential bias caused by tissue-specific transcripts in understanding the regulation of Ψ sites, we used only genes expressed in all the samples and/or tissues to compare Ψ modifications among tissues; no sample-specific and/or tissue-specific genes were considered in the analysis. In addition, we calculated the modification ratio but not coverage of the site, so the expression level of the gene did not affect the modification ratio. Noticeably, the average Ψ fraction in mRNA isolated from different tissues varies in both *Arabidopsis* (Fig. 5b) and rice (Extended Data Fig. 9b), implying tissue-specific mRNA Ψ modifications.

Given that the Ψ to U ratio and Ψ site number vary among tissues, we asked whether mRNA Ψ sites could be differentially modified in tissues and potentially distinguish tissue type in plants. We analysed differentially modified and commonly shared Ψ sites among all the tissues in rice and *Arabidopsis*, respectively. A total of 54 and 122 shared Ψ sites were identified among all rice and *Arabidopsis* tissues (Fig. 5c,d). By contrast, larger numbers of tissue differentially modified Ψ sites were identified in mRNAs from rice and *Arabidopsis* when compared with shared sites (Fig. 5c,d). This observation may suggest roles for differentially modified Ψ in regulating tissue differentiation and development. For example, in *Arabidopsis*, mRNA with seed-specific Ψ sites is markedly enriched in the seed maturation pathway, including the genes *AT1G29760*, *AT1G48130*, *AT2G38560*, *AT4G02280*, *AT4G28520*, *AT5G44120* and *AT5G53470* (Fig. 5e). Meanwhile, transcripts modified with specific Ψ sites in embryo are markedly enriched during post-embryonic plant organ development in rice (Fig. 5f). Note that we observed greater fractions of tissue shared Ψ sites than tissue differentially modified sites (Extended Data Fig. 9c,d), probably indicating important roles for these shared Ψ sites among tissues in maintaining plant transcriptome metabolism across the entire life cycle.

### Ψ correlates with mRNA stability and translation efficiency

**Fig. 6 |:**
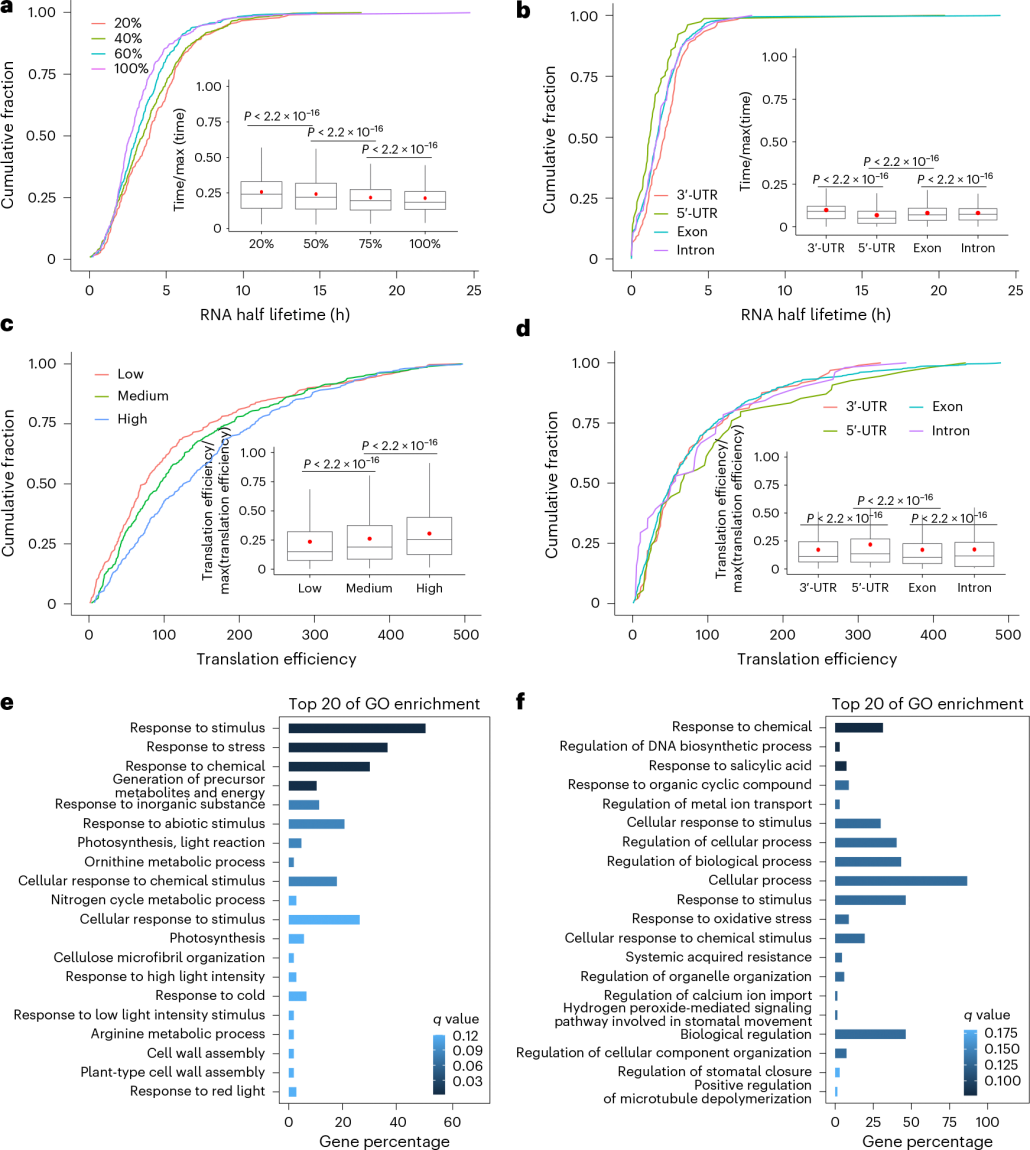
Ψ modifications affect mRNA stability and translation in *Arabidopsis* seedlings. **a**, Cumulative curves and boxplots showing mRNA lifetime distribution for transcripts with Ψ modification. A total of 1,801 transcripts were split into four equal-sized groups based on their quantiles. **b**, Cumulative curves and boxplots showing mRNA lifetime distribution for transcripts with Ψ modification in the 3′-UTR (*n* = 132), 5′-UTR (*n* = 73), exon (*n* = 1,517) and intron (*n* = 79). **c**, Ψ fractions show strong positive correlations with translation efficiency. Translation efficiency was calculated based on polysome profiling data in *Arabidopsis* seedlings. Transcripts were grouped into three categories, low (*n* = 622), medium (*n* = 619) and high (*n* = 520), based on the sum of the Ψ fraction. **d**, Ψ locations in gene regions have different effects on translation efficiency. Translation efficiency was calculated by polysome profiling data in *Arabidopsis* seedlings. Transcript regions of 3′-UTR (*n* = 132), 5′-UTR (*n* = 73), exon (*n* = 1,517) and intron (*n* = 79) are shown. Red dots indicate the mean, lines show the median, boxes represent the IQR and whiskers extend to 1.5× IQR. **e**, GO enrichment analysis of 3′-UTR Ψ-associated mRNAs. **f**, GO enrichment analysis of 5′-UTR Ψ-associated mRNAs. The *Arabidopsi*s seedling lifetime dataset GSE118462 was used for mRNA decay analysis. The *P* value was determined by one-tailed Wilcoxon rank-sum test.

mRNA pseudouridylation has been reported to increase the lifetime and translation of target mRNA in mammals^42^ and parasites^17^. Ψ installed by the pseudouridylation writer TRUB1 stabilizes its target mRNA in mammals^10^. However, the effects of pseudouridylation in plant tissues have yet to be examined. We retrieved publicly available mRNA lifetime data for 7-day-old *Arabidopsis* seedlings^43^, which are at the same growth stage as the Ψ profile of *Arabidopsis* seedlings in this study. To explore how Ψ fraction may correlate with mRNA stability, we divided mRNA carrying Ψ into four groups based on their integrated fractions for the individual transcripts in seedlings. We found that higher Ψ fractions are associated with lower mRNA stability in these transcripts (Fig. 6a). We then asked whether the position of Ψ sites could influence mRNA stability^44,45,46^. To answer this, we first clustered Ψ sites into groups of 3′-UTR Ψ, 5′-UTR Ψ, exon Ψ and intron Ψ, and compared the mRNA lifetime distribution. We observed that transcripts modified with 5′-UTR Ψ sites show low lifetime distribution in comparison with transcripts modified with 3′-UTR Ψ (Fig. 6b). Therefore, it is probable that 5′-UTR Ψ sites have a more important role in destabilizing mRNA in *Arabidopsis* seedlings.

We next explored the correlation between Ψ and translation in mRNA using the corresponding polysome datasets from *Arabidopsis* seedlings. To our surprise, we observed a positive correlation between Ψ level and translation efficiency in transcripts modified with Ψ (Fig. 6c). We also found that transcripts bearing 5′-UTR Ψ sites correlate more significantly with increased translation efficiency compared with transcripts with non-5′-UTR Ψ sites (Fig. 6d), contradicting the correlation observed for mRNA decay. Taken together, these observations suggest rather complex roles for Ψ modifications in regulating mRNA decay and translation in plants, which are affected by both the location and fraction of modified Ψ sites. The complex effects of Ψ sites in the 5′-UTR and 3′-UTR on controlling mRNA stability and translation prompted us to investigate whether the location of Ψ also correlates with different biological functions. Gene Ontology (GO) enrichment analysis showed that genes associated with either the 3′-UTR Ψ (Fig. 6e) or 5′-UTR Ψ (Fig. 6f) are enriched in stimulus responses; however, 3′-UTR Ψ-bearing mRNAs are specifically enriched in photosynthesis-related pathways in *Arabidopsis* (Fig. 6e).

## Discussion

Benefit from the advancements in quantitative sequencing methods for Ψ^13,29^, here, we report comprehensive maps of Ψ at single-base precision with stoichiometry information in ten rice tissues and nine *Arabidopsis* tissues spanning their life cycles, as well as shoots of maize and soybean. We provide in-depth resources for future investigations into Ψ functions of the whole transcriptome in the plant kingdom. We found that although rRNA and tRNA Ψ sites tend to be more conserved between plant species and among different plant tissues, their stoichiometry appears to be critical to translation regulation. By contrast, plant mRNA Ψ sites have more tissue-specific distribution, which may help regulate tissue-specific differentiation and development events.

mRNA levels do not simply reflect protein translation efficiency, with diverse features on rRNA, tRNA and mRNA all playing complex roles. Ψ is frequently distributed in evolutionarily conserved regions of non-coding RNA in mammals^47^. Ψ modification loss in the rRNA A-loop of the peptidyl transferase centre could lead to a substantial decrease in translation activity in yeast^48^. A Ψ site in the T-arm of tRNA regulates translation in *Escherichia coli*^41^. These results highlight the importance of Ψ in rRNA and tRNA for the regulation of translation efficiency in non-plant species. Here, we unveil a multilayered model of translation regulation through Ψ modifications on rRNA, tRNA and mRNA in plants. Ψ sites deposited in rRNA have a higher density of modifications in conserved regions than in variable regions in both plant species and mammals. We uncover evolutionary conservation of rRNA Ψ sites between monocots and dicots. Combining Ψ modifications with RNA abundance and polysome profiling data, we noticed that high Ψ stoichiometry on mRNA correlates with high translation efficiency and probably acts as a contributor to tissue-selective translation control in *Arabidopsis*. Features of the 5′-UTR, such as length, can have particular roles on translation efficiency control^49^. We observed that gene transcripts with a longer 5′-UTR show more positive correlation between their translation efficiency and Ψ level for rRNA Ψ sites at the ribosomal initiation centre, and gene transcripts with a longer CDS show more positive correlation between their translation efficiency and Ψ level for rRNA Ψ site at the ribosomal decoding centre. Although the rRNA Ψ level could control translation globally, stronger effects were observed on transcripts with a long 5′-UTR or CDS, correlating with Ψ deposition at either the ribosomal initiation or decoding centre, respectively.

In tRNAs, modifications in the anticodon stem-loop are generally considered to have direct roles during translation; however, the functional effects on translation of modifications like Ψ in the rest of tRNA remain elusive. Our analysis uncovered that the tRNA Ψ modification stoichiometry, especially for the tRNA loop regions, positively correlates with the translation efficiency of their respective codons, although no strong correlation was observed for tRNA elbow regions. To our surprise, we found that the correlation in the T-arm loop is much stronger than in the anticodon-arm loop, at least for Ψ modification. This might suggest that Ψ modification in the T-arm loop is critical for the tRNA molecule to modulate its recognition by ribosomes, becoming a requisite to guide translation through the anticodon-arm loop. This pathway offers a new layer of global translation tuning through tRNA pseudouridylation. Therefore, Ψ modification on both rRNA and tRNA can impact the translation machinery, offering broad-scale regulation of the transcriptome. rRNA Ψ modifications globally regulate translation and the stoichiometry at individual sites can vary in translation control, whereas tRNA Ψ modifications in the loop region might be critical in translation efficiency control.

Our analysis also revealed prevalent Ψ sites in plant mRNA across different plant species and tissue types, although the average modification fraction in plant tissues is lower than that in mouse tissues. Different from rRNA and tRNA, we uncovered a large portion of tissue-specific mRNA Ψ sites. In comparison with tissue-shared mRNA Ψ sites, the differentially modified Ψ sites in various tissues are probably important in tissue-specific differentiation and development functions across plant life cycle. Although Ψ modifications on mRNA are abundant in all the plant species and tissues we tested, our analysis revealed that mRNA Ψ modifications are not evolutionarily conserved between plant species, further supporting plant- and tissue-specific functions of mRNA pseudouridylation.

Ψ extends the half-life of mRNA and complete substitution of uridines in mRNA with Ψ results in higher translation efficiency in human cells^23^. The replacement of uridines with Ψ in mRNA might lead to a more structured CDS that could, in part, stabilize mRNA in human cells^23^. However, mRNA Ψ may lead to mixed effects on translation that depend on the sequence context and local secondary structures^50,51^. We observed a complex effect of Ψ on target mRNA in *Arabidopsis*. The extent of the mRNA stabilization effect is negatively correlated with Ψ fraction such that a high Ψ fraction is linked to short lifetime in *Arabidopsis* seedlings. When examining the correlation between Ψ modification and translation efficiency, the Ψ fraction in mRNA is positively correlated with translation efficiency in *Arabidopsis* seedlings. Therefore, it is probable that Ψ-modified mRNA in *Arabidopsis* negatively affects mRNA stability, but the reduced stability may not necessarily cause reduced translation efficiency in *Arabidopsis* seedlings, warranting future investigation of individual pseudouridine synthetase and individual Ψ sites. Our findings highlight the importance of Ψ modification stoichiometry on rRNA, tRNA and mRNA contributing to diverse translation control in different plant tissues.

## Methods

### Plant material

For *Arabidopsis*, Col-0 accession was used in this study. Plants were grown at 20 °C with 16 h of light and 8 h of dark on 1/2 Murashige and Skoog medium (MS) plates. Seedlings were collected after growing for 7 days, whereas shoots and roots were collected separately after growing for 14 days. Plants were then transplanted to soil. Rosetta leaves were collected after growing for 30 days, whereas cauline leaf, flower, stem and silique were collected after flowering. *Arabidopsis* seeds were collected once they were completely mature and dried. For rice, Nipponbare was used in this study. Plants were grown at 28 °C with 14 h of light and 10 h of dark on 1/2 MS plates. Rice plumule and radicle were collected on 1/2 MS plates 48 h after germination. Rice plants were then transplanted to soil. Next, 8-day-old and 2-week-old seedlings were collected accordingly. Straw, flag leaf and panicles were collected at the heading stage. At 10 days after anthesis, flag leaf, endosperms and embryos were collected. Maize inbred line B73 seeds and soybean Williams 82 (W82) seeds were germinated in soil at 28 °C under 14 h of light per 24 h. Shoots of 7-day-old maize and soybean plants were harvested. Total RNA was extracted using TRIzol Reagent (Invitrogen, catalogue number 15596026) according to the manufacturer’s instructions. All plants were planted in the greenhouse of the University of Chicago.

### The *osrlua3* and *svr1* mutants in rice

We bought *osrlua3* mutant (BG101402C11) in the ZhongHua11 (ZH11) background and *svr1* mutant (BG101343B10) in the Nipponbare background from Biogle. CCAGGTCACTCACCCTCCCTCCAT in LOC_Os07g46600 and GATGTTGAAGTCCGGGGCGCTGG in LOC_Os03g05806 were selected, respectively, as the target sequence for the construction of *osrlua3* and *svr1* mutants. The forward primer of TGCGGTGTCCACCTCCG and reverse primer of CGTTTGGGATGCACATGAACC were used to verify mutation of the *osrlua3* mutant. The forward primer of GATAAACCCCCTCCCACACTCT and reverse primer of AAGGACCTTGGCGAGCC were used to verify the *svr1* mutant.

### mRNA capture from the extracted total RNA

We used 50 μg of total RNA for mRNA capture (Invitrogen, Dynabeads mRNA DIRECT Purification Kit, catalogue number 61012) for each biological replicate. Total RNA was denatured by incubating at 65 °C for 2 min in a 100 μl volume (dilute by RNase-free water), and then immediately moved onto ice for 2 min. We used 100 μl of Dynabeads for each sample for the mRNA capture procedure by washing twice with 200 μl of lysis/binding buffer provided in the above kit. The washed beads were then resuspended with 100 μl of lysis/binding buffer and mixed with the above denatured total RNA. The mixture was incubated on a rotor for 15 min at room temperature. After binding, beads were washed twice with buffer B (provided with the kit) and then eluted using 30 μl of H_2_O at 75 °C for 2 min. Next, the eluted mRNA collected from the beads was purified with one round of poly(A) enrichment and used in the sequencing library construction. One round of poly(A) enrichment could be used for rRNA Ψ mapping. The leftover rRNA after poly(A) enrichment still yielded sufficiently deep coverage for analysis based on comparison of total RNA BID-seq and one round poly(A) enrichment RNA BID-seq.

### Quantification of Ψ in RNA by LC–MS/MS

The mRNA capture process was repeated twice, as described above, to obtain more purified mRNA for LC–MS/MS. Fifty nanograms of poly(A)-enriched mRNA was digested into nucleosides, and the amount of Ψ was measured using a ZORBAX SB-Aq 4.6 × 50 mm column (Agilent, catalogue number AG835975) on ultra high-performance liquid chromatography coupled to a triple quadrupole mass spectrometer (Agilent 6460 Series Triple Quad MS-MS with 1290 Infinity Series LC). For each sample, RNA was digested using nuclease P1 (NEB) at 37 °C for 2 h. Then, 1 μl of shrimp alkaline phosphatase (NEB, catalogue number M0371L) and 3 μl of 10× rCutsmart buffer (NEB, catalogue number B6004S) was added, and the reaction was incubated at 37 °C for 2 h. Samples were then filtered through a 0.22 μm filter (Millipore, catalogue number GSWP04700) and injected into LC–MS/MS. Nucleosides were quantified using the nucleoside-to-base ion mass transitions of Ψ (245.1 to 125) to U (245 to 113.1) and compared with calibration curves. Three biological independent replicates were used for Ψ level quantification, and each sample was injected three times. The ratio of Ψ to U was calculated based on the calibrated concentrations. Note that we used RNA modifications (m^2^_2_G, m^2^G, m^6^_2_A and m^1^A), existing only on rRNA or tRNA species, to verify that two rounds of poly(A) enrichment could remove most non-poly(A) RNA species.

### BID-seq library construction

Fifty nanograms of mRNA (purified with one round of poly(A) enrichment) from each replicate was used for the library construction. Although for the total RNA of the nine *Arabidopsis* tissues, 100 ng of total RNA of each replicate was used. All libraries were constructed exactly following previously published protocols^29^. The constructed libraries were sequenced on the Illumina NovaSeq sequencing platform in single read mode with 100 bp per read.

### BID-seq data processing

After sequencing, the Ψ sites were detected using a previously published method (https://github.com/y9c/pseudoU-BIDseq). The analysis utilized the reference genome downloaded from the Ensemble database, with assembly versions TAIR10, IRGSP-1.0, Glycine_max_v2.1 and Zm-B73-REFERENCE-NAM-5.0 used for *Arabidopsis*, rice, soybean and maize, respectively. Replicates from one tissue are combined to calculate the Ψ stoichiometry. Briefly, gapped and non-gapped read counts for each site were summed, and the gap ratio was calculated as the proportion of gapped read counts over total read counts at each site. The gap ratio was then calibrated by fitting a calibration curve to calculate the Ψ stoichiometry. The high-quality sites were filtered using a *P* value-based method, as described previously^13^.

### Polysome profiling library construction

Polysome profiling was conducted with the nine *Arabidopsis* tissues based on a previously published sucrose gradient method^44^. Briefly, we first prepared the sucrose gradient buffer: 60 ml of 5% sucrose (5 ml of 60% sucrose + 49 ml of H_2_O + 6 ml of 10X buffer) and 50% sucrose (50 ml of 60% sucrose + 4 ml of H_2_O + 6 ml of 10X buffer) were each prepared in 1× sucrose gradient buffer + 100 μg ml^−1^ cycloheximide (C1988), 0.1% protease inhibitor and 0.1% SUPERaseIn (Invitrogen, catalogue number AM2696). Half of the 50% sucrose buffer was then placed at the bottom of a high-speed centrifuge tube (SETON 7042), half of the 5% sucrose buffer was placed above it and gradient sucrose buffer was made using a BioComp Gradient station. For sample preparation, lysis buffer with 20 mM HEPES, pH 7.6, 100 mM KCl, 5 mM MgCl_2_, 100 μg ml^−1^ cycloheximide, 1% Triton X-100, freshly added 1:100 protease inhibitor (Roche) and 40 U ml^−1^ SUPERaseIn was added to 0.3 g of freshly ground nine *Arabidopsis* samples with two biological replicates. After 30 min of lysis, samples were centrifuged at 16,000*g* for 15 min and the clear lysate was saved for further use. Then, 5 μl of Turbo DNase was added to 1 ml of the above lysate and incubated at room temperature for 15 min. The lysate was then carefully placed on top of the gradient sucrose buffer and centrifuged at 104,000*g* for 3 h at 4 °C. After centrifugation, we collected the fraction using the BioComp Gradient station and tubes 6, 7, 8 and 9–14 were collected. Finally, the collected tubes were mixed equally for further library preparation. The library preparation procedures were as follows. We first measured the optical density at 260 nm for the mixed fraction (300 μl) of each sample described in the last step and then added RNase I (that is, optical density = 1, add 2.7 U of RNase I), followed by incubation at 37 °C for 30 min. Three volumes of LS TRIzol were then added to the samples and mixed well for RNA extraction. Next, 300 μl of chloroform was added and mixed well for 5 min. The samples were then centrifuged for 15 min to collect the supernatant into a new 1.5 ml tube. Equal volumes of 2-propanol and 1 μl of GlycoBlue (AM9516) were added to the above supernatant and the mixture was kept at −80 °C overnight for RNA precipitation. After centrifuging for 15 min, the precipitation was washed with 70% EtOH and 15 μl of RNase-free H_2_O was added to resolve the RNA. The RNA was then run in 15% ten-well TBE-Urea Gel (Invitrogen, catalogue number EC6885BOX) to select RNA fragments between 20 and 40 nucleotides in size (prestained ladder (DM253S) was used for size reference). Libraries were then constructed following the procedures given in the preparation kit (NEB, catalogue number E7330S). The constructed libraries were sequenced on an Illumina NovaSeq sequencing platform in single read mode with 100 bp per read.

### Polysome profiling data processing

Sequencing data with read lengths between 29 and 35 bp were selected and mapped to the *Arabidopsis* reference genome (TAIR10) using HISAT2 aligner. After mapping, coverage at each position along the open reading frames was extracted using Ribotricer (https://github.com/smithlabcode/ribotricer), applying the recommended phase-score cut-off for *Arabidopsis* and default settings. Codon translation efficiency was defined as the reciprocal of the dwell time for each codon, calculated from the relative coverage of codons obtained from ribosome footprinting, and normalized by gene expression levels derived from RNA-seq.

### Correlation of Ψ stoichiometry with translation efficiency

Translation efficiencies were quantified at both gene (transcript) and codon level, and noted as *E*_transcript protein abundance_, *E*_transcript_ and *E*_codon_, respectively. Protein abundance aligns with gene expression level in general, but is affected by post-transcriptional regulation processes, such as RNA modification. The ratio of protein abundance to RNA abundance reflects the translation efficiency at the transcript level (*E*_transcript protein abundance_). Data for protein and RNA abundance at different tissues were downloaded from previous research^33^. In this study, intensity-based absolute quantification was used as the measure of protein levels, and transcripts per kilobase million (TPM) was used as the measure of RNA levels. We processed the data following the same approach^33^. We also calculated the translation efficiency for each gene by normalizing the ribosome-bound RNA level (with the polysome footprinting datasets) to the RNA level (*E*_transcript_). Translation efficiency can also be measured at the codon level and calculated using polysome footprinting data. Codon translation efficiency is regarded as a reciprocal of the dwell time of each codon, and was calculated using the relative coverage of codon from ribosome footprinting and normalized by gene expression level from RNA-seq.

### Identification of orthologous genes among four plant species

All Ensembl orthologous gene pairs were downloaded from the Plant Homologues database, using all annotated genes in the *Arabidopsis* TAIR10 database as a reference (*N* = 32,833). Specifically, 19,368 genes in maize, 15,039 genes in rice and 33,335 genes in soybean were identified as one-to-one or one-to-many orthologues, resulting in 37,294, 29,246 and 52,511 orthologous gene pairs, respectively. All orthologous genes were aligned to identify homologous sites using the Smith–Waterman algorithm. Site pairs can be categorized into four classes: Class 1, non-homologous sites (sites that do not show homologous sites in another species); Class 2, non-conserved homologous sites (sites that show homologous sites in another species, but not both sites are U); Class 3, one-to-one conserved sites (sites that have homologous sites in another species, with U conserved in both species); and Class 4, one-to-many conserved sites (sites that show multiple homologous U sites in another species). Classes 3 and 4 are defined as conserved sites, whereas classes 1 and 2 are defined as non-conserved sites.

### GO analysis

Functional GO enrichment analysis was performed using a web-based toolkit for the agricultural community agriGO v.2.038 (http://systemsbiology.cau.edu.cn/agriGOv2/). GO terms with a false discovery rate <0.05 were considered significantly enriched.

### Statistics and reproducibility

All experiments were repeated independently at least twice and showed similar results. GraphPad Prism v.9 and R studio were used for the figure plotting.

## Extended Data

## Supplementary Material

Supplementary Tables

Source Data

**Supplementary information** The online version contains supplementary material available at https://doi.org/10.1038/s41477-024-01894-7.

## Data Availability

All data supporting the findings of this study are available in the main text or the Supplementary tables. The BID-seq and RNA-seq data reported in this study have been deposited in the Gene Expression Omnibus database (https://www.ncbi.nlm.nih.gov/geo) under accession numbers GSE262373, GSE262374, GSE262375, GSE262376, GSE277198 and GSE277201. Source data are provided with this paper.

## References

[1] Cohn WE 5-Ribosyl uracil, a carbon–carbon ribofuranosyl nucleoside in ribonucleic acids. Biochim. Biophys. Acta 32, 569–571 (1959). 13811055 10.1016/0006-3002(59)90644-4

[2] Hamma T & Ferre-D’Amare AR Pseudouridine synthases. Chem. Biol. 13, 1125–1135 (2006). 17113994 10.1016/j.chembiol.2006.09.009

[3] Spenkuch F, Motorin Y & Helm M Pseudouridine: still mysterious, but never a fake (uridine)! RNA Biol. 11, 1540–1554 (2014). 25616362 10.4161/15476286.2014.992278 PMC4615568

[4] Li X, Ma S & Yi C Pseudouridine: the fifth RNA nucleotide with renewed interests. Curr. Opin. Chem. Biol. 33, 108–116 (2016). 27348156 10.1016/j.cbpa.2016.06.014

[5] De Zoysa MD & Yu YT Posttranscriptional RNA pseudouridylation. Enzymes 41, 151–167 (2017). 28601221 10.1016/bs.enz.2017.02.001 PMC5694665

[6] Adachi H, De Zoysa MD & Yu YT Post-transcriptional pseudouridylation in mRNA as well as in some major types of noncoding RNAs. Biochim. Biophys. Acta Gene Regul. Mech. 1862, 230–239 (2019). 30414851 10.1016/j.bbagrm.2018.11.002 PMC6401265

[7] Westhof E Pseudouridines or how to draw on weak energy differences. Biochem. Biophys. Res. Commun. 520, 702–704 (2019). 31761086 10.1016/j.bbrc.2019.10.009

[8] Carlile TM Pseudouridine profiling reveals regulated mRNA pseudouridylation in yeast and human cells. Nature 515, 143–146 (2014). 25192136 10.1038/nature13802 PMC4224642

[9] Lovejoy AF, Riordan DP & Brown PO Transcriptome-wide mapping of pseudouridines: pseudouridine synthases modify specific mRNAs in *S. cerevisiae*. PLoS ONE 9, e110799 (2014). 25353621 10.1371/journal.pone.0110799 PMC4212993

[10] Schwartz S Transcriptome-wide mapping reveals widespread dynamic-regulated pseudouridylation of ncRNA and mRNA. Cell 159, 148–162 (2014). 25219674 10.1016/j.cell.2014.08.028 PMC4180118

[11] Li X Chemical pulldown reveals dynamic pseudouridylation of the mammalian transcriptome. Nat. Chem. Biol. 11, 592–597 (2015). 26075521 10.1038/nchembio.1836

[12] Roovers M Formation of the conserved pseudouridine at position 55 in archaeal tRNA. Nucleic Acids Res. 34, 4293–4301 (2006). 16920741 10.1093/nar/gkl530 PMC1616971

[13] Zhang LS BID-seq for transcriptome-wide quantitative sequencing of mRNA pseudouridine at base resolution. Nat. Protoc. 19, 517–538 (2024). 37968414 10.1038/s41596-023-00917-5 PMC11007761

[14] Carlile TM mRNA structure determines modification by pseudouridine synthase 1. Nat. Chem. Biol. 15, 966–974 (2019). 31477916 10.1038/s41589-019-0353-z PMC6764900

[15] Sun L Transcriptome-wide analysis of pseudouridylation of mRNA and non-coding RNAs in *Arabidopsis*. J. Exp. Bot. 70, 5089–5600 (2019). 31173101 10.1093/jxb/erz273 PMC6793436

[16] Krutyholowa R, Zakrzewski K & Glatt S Charging the code – tRNA modification complexes. Curr. Opin. Struct. Biol. 55, 138–146 (2019). 31102979 10.1016/j.sbi.2019.03.014

[17] Nakamoto MA, Lovejoy AF, Cygan AM & Boothroyd JC mRNA pseudouridylation affects RNA metabolism in the parasite *Toxoplasma gondii*. RNA 23, 1834–1849 (2017). 28851751 10.1261/rna.062794.117 PMC5689004

[18] Sloan KE Tuning the ribosome: the influence of rRNA modification on eukaryotic ribosome biogenesis and function. RNA Biol. 14, 1138–1152 (2017). 27911188 10.1080/15476286.2016.1259781 PMC5699541

[19] Jack K rRNA pseudouridylation defects affect ribosomal ligand binding and translational fidelity from yeast to human cells. Mol. Cell 44, 660–666 (2011). 22099312 10.1016/j.molcel.2011.09.017 PMC3222873

[20] Pederiva C Control of protein synthesis through mRNA pseudouridylation by dyskerin. Sci. Adv. 9, eadg1805 (2023). 37506213 10.1126/sciadv.adg1805 PMC10381945

[21] Basu A Requirement of rRNA methylation for 80S ribosome assembly on a cohort of cellular internal ribosome entry sites. Mol. Cell. Biol. 31, 4482–4499 (2011). 21930789 10.1128/MCB.05804-11 PMC3209261

[22] Zhao Y, Rai J & Li H Regulation of translation by ribosomal RNA pseudouridylation. Sci. Adv. 9, eadg8190 (2023). 37595043 10.1126/sciadv.adg8190 PMC10438446

[23] Eyler DE Pseudouridinylation of mRNA coding sequences alters translation. Proc. Natl Acad. Sci. USA 116, 23068–23074 (2019). 31672910 10.1073/pnas.1821754116 PMC6859337

[24] Niu Y The *Arabidopsis* mitochondrial pseudouridine synthase homolog FCS1 plays critical roles in plant development. Plant Cell Physiol. 63, 955–966 (2022). 35560171 10.1093/pcp/pcac060

[25] Lu S, Li C, Zhang Y, Zheng Z & Liu D Functional disruption of a chloroplast pseudouridine synthase desensitizes *Arabidopsis* plants to phosphate starvation. Front. Plant Sci. 8, 1421 (2017). 28861101 10.3389/fpls.2017.01421 PMC5559850

[26] Wang Z Pseudouridylation of chloroplast ribosomal RNA contributes to low temperature acclimation in rice. New Phytol. 236, 1708–1720 (2022). 36093745 10.1111/nph.18479

[27] Fleming AM Structural elucidation of bisulfite adducts to pseudouridine that result in deletion signatures during reverse transcription of RNA. J. Am. Chem. Soc. 141, 16450–16460 (2019). 31538776 10.1021/jacs.9b08630 PMC6817977

[28] Khoddami V Transcriptome-wide profiling of multiple RNA modifications simultaneously at single-base resolution. Proc. Natl Acad. Sci. USA 116, 6784–6789 (2019). 30872485 10.1073/pnas.1817334116 PMC6452723

[29] Dai Q Quantitative sequencing using BID-seq uncovers abundant pseudouridines in mammalian mRNA at base resolution. Nat. Biotechnol. 41, 344–354 (2023). 36302989 10.1038/s41587-022-01505-w PMC10017504

[30] Taoka M Landscape of the complete RNA chemical modifications in the human 80S ribosome. Nucleic Acids Res. 46, 9289–9298 (2018). 30202881 10.1093/nar/gky811 PMC6182160

[31] Taoka M A mass spectrometry-based method for comprehensive quantitative determination of post-transcriptional RNA modifications: the complete chemical structure of *Schizosaccharomyces pombe* ribosomal RNAs. Nucleic Acids Res. 43, e115 (2015). 26013808 10.1093/nar/gkv560 PMC4605285

[32] Ofengand J & Bakin A Mapping to nucleotide resolution of pseudouridine residues in large subunit ribosomal RNAs from representative eukaryotes, prokaryotes, archaebacteria, mitochondria and chloroplasts. J. Mol. Biol. 266, 246–268 (1997). 9047361 10.1006/jmbi.1996.0737

[33] Mergner J Mass-spectrometry-based draft of the *Arabidopsis* proteome. Nature 579, 409–414 (2020). 32188942 10.1038/s41586-020-2094-2

[34] Arribere JA & Gilbert WV Roles for transcript leaders in translation and mRNA decay revealed by transcript leader sequencing. Genome Res. 23, 977–987 (2013). 23580730 10.1101/gr.150342.112 PMC3668365

[35] Davyt M, Bharti N & Ignatova Z Effect of mRNA/tRNA mutations on translation speed: implications for human diseases. J. Biol. Chem. 299, 105089 (2023). 37495112 10.1016/j.jbc.2023.105089 PMC10470029

[36] Cui W tRNA modifications and modifying enzymes in disease, the potential therapeutic targets. Int. J. Biol. Sci. 19, 1146–1162 (2023). 36923941 10.7150/ijbs.80233 PMC10008702

[37] Liu Y tRNA-m(1)A modification promotes T cell expansion via efficient MYC protein synthesis. Nat. Immunol. 23, 1433–1444 (2022). 36138184 10.1038/s41590-022-01301-3

[38] Zaborske JM A nutrient-driven tRNA modification alters translational fidelity and genome-wide protein coding across an animal genus. PLoS Biol. 12, e1002015 (2014). 25489848 10.1371/journal.pbio.1002015 PMC4260829

[39] Song J Differential roles of human PUS10 in miRNA processing and tRNA pseudouridylation. Nat. Chem. Biol. 16, 160–169 (2020). 31819270 10.1038/s41589-019-0420-5

[40] Agris PF Bringing order to translation: the contributions of transfer RNA anticodon-domain modifications. EMBO Rep. 9, 629–635 (2008). 18552770 10.1038/embor.2008.104 PMC2475317

[41] Schultz SK Modifications in the T arm of tRNA globally determine tRNA maturation, function, and cellular fitness. Proc. Natl Acad. Sci. USA 121, e2401154121 (2024). 38889150 10.1073/pnas.2401154121 PMC11214086

[42] Kariko K Incorporation of pseudouridine into mRNA yields superior nonimmunogenic vector with increased translational capacity and biological stability. Mol. Ther. 16, 1833–1840 (2008). 18797453 10.1038/mt.2008.200 PMC2775451

[43] Szabo EX Metabolic labeling of RNAs uncovers hidden features and dynamics of the *Arabidopsis* transcriptome. Plant Cell 32, 871–887 (2020). 32060173 10.1105/tpc.19.00214 PMC7145469

[44] Shi H YTHDF3 facilitates translation and decay of N(6)-methyladenosine-modified RNA. Cell Res. 27, 315–328 (2017). 28106072 10.1038/cr.2017.15 PMC5339834

[45] He PC & He C m(6) A RNA methylation: from mechanisms to therapeutic potential. EMBO J. 40, e105977 (2021). 33470439 10.15252/embj.2020105977 PMC7849164

[46] Hu J N(6)-Methyladenosine mRNA methylation is important for salt stress tolerance in *Arabidopsis*. Plant J. 106, 1759–1775 (2021). 33843075 10.1111/tpj.15270

[47] Hsu PJ, Shi H & He C Epitranscriptomic influences on development and disease. Genome Biol. 18, 197 (2017). 29061143 10.1186/s13059-017-1336-6 PMC5654102

[48] Liang XH, Liu Q & Fournier MJ Loss of rRNA modifications in the decoding center of the ribosome impairs translation and strongly delays pre-rRNA processing. RNA 15, 1716–1728 (2009). 19628622 10.1261/rna.1724409 PMC2743053

[49] Mignone F, Gissi C, Liuni S & Pesole G Untranslated regions of mRNAs. Genome Biol. 3, REVIEWS0004 (2002). 11897027 10.1186/gb-2002-3-3-reviews0004 PMC139023

[50] Hoernes TP Eukaryotic translation elongation is modulated by single natural nucleotide derivatives in the coding sequences of mRNAs. Genes 10, 84 (2019). 30691071 10.3390/genes10020084 PMC6409545

[51] Hoernes TP Nucleotide modifications within bacterial messenger RNAs regulate their translation and are able to rewire the genetic code. Nucleic Acids Res. 44, 852–862 (2016). 26578598 10.1093/nar/gkv1182 PMC4737146

